# Deletion of *Mgat2* in spermatogonia blocks spermatogenesis

**DOI:** 10.3389/fcell.2024.1428715

**Published:** 2024-09-19

**Authors:** Mohd Shamoon Asmat, Xiang Yu Zheng, Mohd Nauman, Deyou Zheng, Pamela Stanley

**Affiliations:** ^1^ Department of Cell Biology, Albert Einstein College of Medicine, New York, NY, United States; ^2^ Department of Genetics, Albert Einstein College of Medicine, New York, NY, United States; ^3^ Department of Neurology and Neuroscience, Albert Einstein College of Medicine, New York, NY, United States

**Keywords:** complex N-glycans, MGAT2, conditional knockout, spermatogenesis, AKT and ERK signaling

## Abstract

Identifying factors required for spermatogenesis is important for understanding mechanisms of male fertility. Inactivation of either the *Mgat1* or *Man2a2* gene leads to a block in spermatogenesis causing infertility in male mice. MGAT1 GlcNAc-transferase initiates complex N-glycan synthesis and MAN2A2 mannosidase generates the substrate for MGAT2 GlcNAc-transferase to form a biantennary complex N-glycan. In this paper, we show that conditional deletion of *Mgat2* in spermatogonia *via Stra8*-iCre caused a novel block in spermatogenesis, largely prior to the formation of round spermatids. *Mgat2*[−/−] germ cells did not bind the lectins *Phaseolus vulgaris* leucoagglutinin (L-PHA) or *Griffonia simplicifolia* II (GSA-II), similar to germ cells lacking MGAT1 and complex N-glycans. However, overall spermatogenic defects were distinct in germ cells with deleted *Mgat2 versus Mgat1*. In addition, RNA-seq analysis at 15 days after birth revealed a unique transcriptomic landscape in *Mgat2*[−/−] germ cells with genes required for sperm formation and functions being most downregulated. Bioinformatic analyses using the ingenuity pathway analysis (IPA) algorithm identified ERK and AKT as central activities. Western blot analyses of 15-day germ cell lysates confirmed that both AKT and ERK1/2 signaling were increased by loss of MGAT2 in germ cells. By contrast, *Mgat1*[−/−] germ cells were previously shown to have reduced ERK signaling and unchanged AKT activity. Therefore, since the loss of all complex N-glycans is common to each mutant model, the different immature N-glycans that accumulate in *Mgat2*[−/−] *versus Mgat1*[−/−] germ cells are proposed to be the basis of their unique spermatogenic phenotypes.

## Introduction

Determining mechanisms necessary for optimal spermatogenesis is important for both understanding male fertility and revealing potential targets for male contraceptives. Our laboratory has focused on defining roles for glycans in mammalian spermatogenesis (Akintayo and Stanley, 2019). We previously showed that conditional knockout (cKO) in spermatogonia (Sg) of the glycosyltransferase gene *C1galt1* (to prevent the synthesis of core 1 and 2 O-GalNAc glycans), or *Pofut1* (to prevent addition of O-fucose glycans to EGF repeats on Notch receptors), or *Mgat1* (to prevent complex N-glycan synthesis) has different outcomes (Batista et al., 2012). Male individuals lacking MGAT1 in germ cells have no sperm whereas loss of C1GALT1 or POFUT1 does not affect spermatogenesis (Batista et al., 2012). This was unexpected since global deletion of *C1galt1* (Xia et al., 2004) or *Pofut1* (Shi and Stanley, 2003; Okamura and Saga, 2008) or *Mgat1* (Ioffe and Stanley, 1994; Metzler et al., 1994) is embryonic lethal.

*Mgat1* cKO in Sg prevents complex N-glycan synthesis in all germ cells and blocks spermatogenesis at the spermatid stage, resulting in infertile male mice (Batista et al., 2012). Transcriptomic analyses indicated defective ERK signaling as a basis of this phenotype, and levels of pERK1 and pERK2 were shown to be reduced in 22-day *Mgat1* cKO germ cells (Biswas et al., 2018; 2023). Global knockout (KO) of *Man2a2*, a mannosidase which removes 2 Man residues from the product of MGAT1 to generate the substrate for the MGAT2 GlcNAc-transferase, also causes male infertility (Akama et al., 2002; Fukuda and Akama, 2002). In this paper, we report the consequences of deleting the next glycosyltransferase in the pathway, *Mgat2,* in spermatogonia. We found that cKO of *Mgat2* via *Stra8-*iCre led to a block in spermatogenesis largely prior to the formation of spermatids, a phenotype distinct from deleting either *Mgat1* or *Man2a2*. Since deletion of either *Mgat1*, *Man2a2*, or *Mgat2* precludes the generation of complex N-glycans, the differing consequences of their deletion in germ cells suggests that their respective phenotypes do not arise solely from the absence of complex N-glycans. RNA-seq comparisons of 15-day *Mgat2* cKO germ cells identified biological processes and molecular networks that provide insights into the spermatogenic defects in *Mgat2* cKO infertile male subjects. In stark contrast to the reduced ERK signaling observed in *Mgat1* cKO germ cells, we show here that 15-day *Mgat2* cKO germ cells exhibited increased signaling of both ERK1/2 and AKT.

## Results

### Mice lacking *Mgat2* in germ cells have a block in spermatogenesis

**FIGURE 1:**
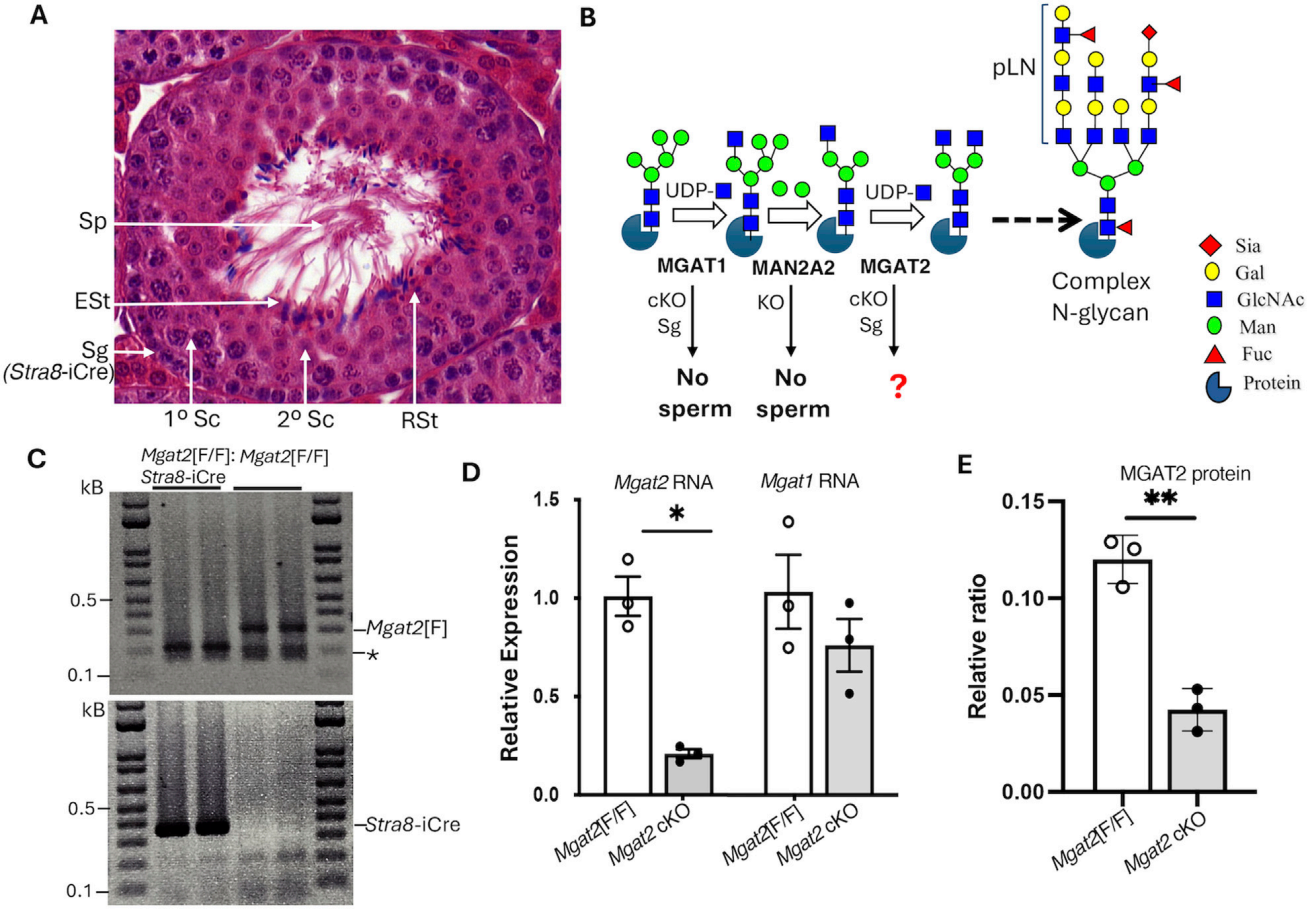
Deletion of *Mgat2* in spermatogonia. **(A)** Diagram showing the different germ cells in a testis tubule from a typical 7-week C57BL6/J male. Sg, spermatogonium; Sc, spermatocyte; RSt, round spermatid; ESt, elongated spermatid; Sp, sperm; *Stra8*-iCre is first expressed in Sg. **(B)** Schematic representation of a portion of the N-glycan biosynthetic pathway showing the reactions catalyzed by MGAT1, MAN2A2, and MGAT2 that precede the generation of branched complex N-glycans and the consequences of deleting *Mgat1* in Sg (cKO) or *Man2a2* globally (KO) (see text for references). Symbols for sugars are from the Symbol Nomenclature for Glycans (Neelamegham et al., 2019). **(C)** PCR genotyping of gDNA from enriched germ cells of 22-day *Mgat2*[F/F] and *Mgat2*[F/F]:*Stra8*-iCre males. * Non-specific band. **(D)** qRT-PCR of cDNA from 22-day *Mgat2*[F/F] and *Mgat2* cKO germ cells to determine expression of *Mgat2* and *Mgat1* transcripts relative to *Actb* and *Gapdh*. **(E)** Western blot analysis of MGAT2 in germ cell lysates of *Mgat2*[F/F] and *Mgat2* cKO 22-day males. The blot quantitated is shown in Supplementary Figure S1C. Histograms are mean ± SEM. Each symbol represents an independent germ cell preparation. *p*-values were determined by Student’s t-test (two-tailed, unpaired) *p < 0.05, **p < 0.01.

Deletion of floxed alleles is achieved in spermatogonia (Sg) with the transgene *Stra8*-iCre, which is expressed 3 days after birth (Figure 1A) (Sadate-Ngatchou et al., 2008). Figure 1B depicts the reactions catalyzed by MGAT1, MAN2A2, and MGAT2 and the consequences of deleting *Mgat1* and *Man2a2* in germ cells. To generate male germ cells lacking *Mgat2*, *Mgat2*[F/F]:*Stra8*-iCre males were produced by mating *Mgat2*[F/F]:*Stra8*-iCre females with *Mgat2*[F/F] males. The average litter size was 9 ± 1 STDEV (n = 10 litters), and the ratio of male to female progeny was not significantly different from 50% (Chi-squared test *p* > 0.5). *Mgat2*[F/F]:*Stra8*-iCre males had testes of reduced size but were otherwise healthy. Testes were characterized at 22 days after birth when tubules contain spermatogonia and spermatocytes but few round spermatids, at 28 days of age when round spermatids predominate and elongated spermatids begin to appear, and at 49 days after birth when all germ cells are present and sperm have formed (Montoto et al., 2012).

To determine the efficiency of deletion of *Mgat2* floxed alleles by *Stra8*-iCre, genomic DNA (gDNA), RNA, and protein were extracted from germ cells of 22-day males. The purity of germ cell preparations was determined by comparing RNA from testis *versus* germ cells from *Mgat2*[F/F] controls. Marked enrichment of germ cells was evident from the low expression of the testis Leydig cell marker *Cyp11a* in germ cell RNA (Supplementary Figure S1A). PCR genotyping showed that floxed *Mgat2* alleles were not detected in *Mgat2*[F/F]:*Stra8*-iCre germ cells (Figure 1C and Supplementary Figure S1B). In addition, *Mgat2* transcripts determined by qRT-PCR were greatly reduced (Figure 1D). Importantly, transcript levels of *Mgat1* were not significantly changed in *Mgat2* cKO germ cells (Figure 1D). MGAT2 protein was also markedly reduced in *Mgat2*[F/F]:*Stra8*-iCre germ cells (Figure 1E and Supplementary Figure S1C), with a low signal presumably contributed by the presence of non-germ cells such as Leydig or Sertoli cells.

**FIGURE 2:**
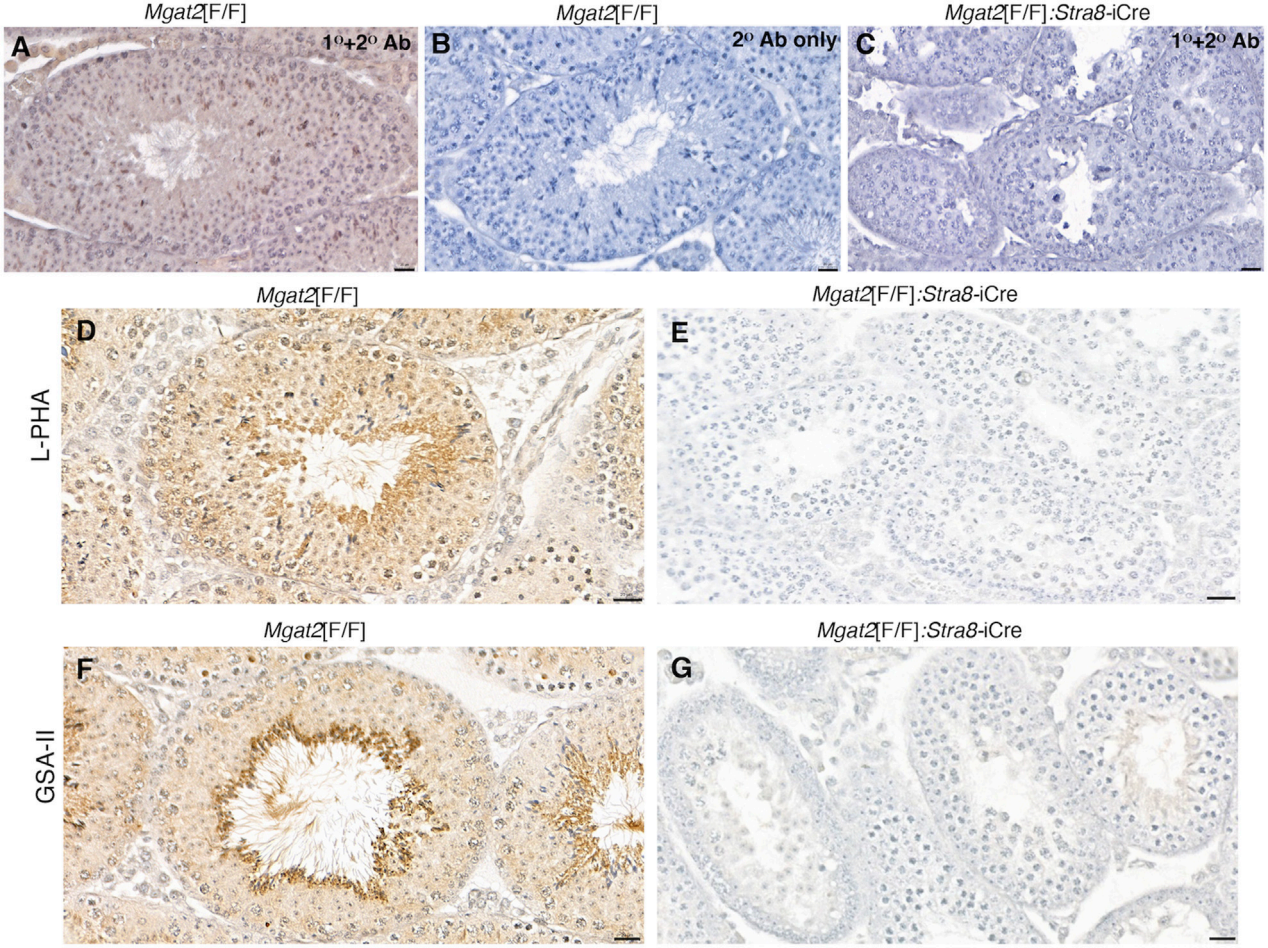
*Mgat2* cKO germ cells lack MGAT2 and complex N-glycans. **(A–C)** Immunohistochemistry of MGAT2 expressed in 7-week *Mgat2*[F/F] and *Mgat2*[F/F]:*Stra8*-iCre testis sections (representative of n = 3 mice per group, two sections per mouse). **(D–G)** Lectin histochemistry with L-PHA and GSA-II in 7-week *Mgat2*[F/F] and *Mgat2*[F/F]:*Stra8*-iCre testis sections shows lectin binding to complex N-glycans only in *Mgat2*[F/F] sections (representative of n = 3 mice per group, two sections per mouse). Scale bars 20 µm. Related low-power images are shown in Supplementary Figure S2. Note that tubule diameters of *Mgat2* cKO testes are smaller than *Mgat2*[F/F] controls.

The loss of MGAT2 from germ cells was confirmed by immunohistochemistry of testis sections from 7-week *Mgat2*[F/F]:*Stra8*-iCre males (Figures 2A–C and Supplementary Figures S2A, S2B). In addition, binding of the plant lectins *Phaseolus vulgaris* leucoagglutinin (L-PHA) and *Griffonia simplicifolia* agglutinin II (GSA-II) to germ cells from the same males was essentially lost, indicating the absence of complex N-glycans (Figures 2D–G and Supplementary Figures S2C–S2F). Both L-PHA and GSA-II bind to the branch sugars of complex N-glycans (Bojar et al., 2022) and do not bind to cells lacking complex N-glycans. Importantly, the lack of L-PHA binding showed that the single GlcNAc on the hybrid N-glycan generated in the absence of MGAT2 was not extended with polylactosamine (pLN) in *Mgat2* cKO germ cells (see Figure 1B).

**FIGURE 3:**
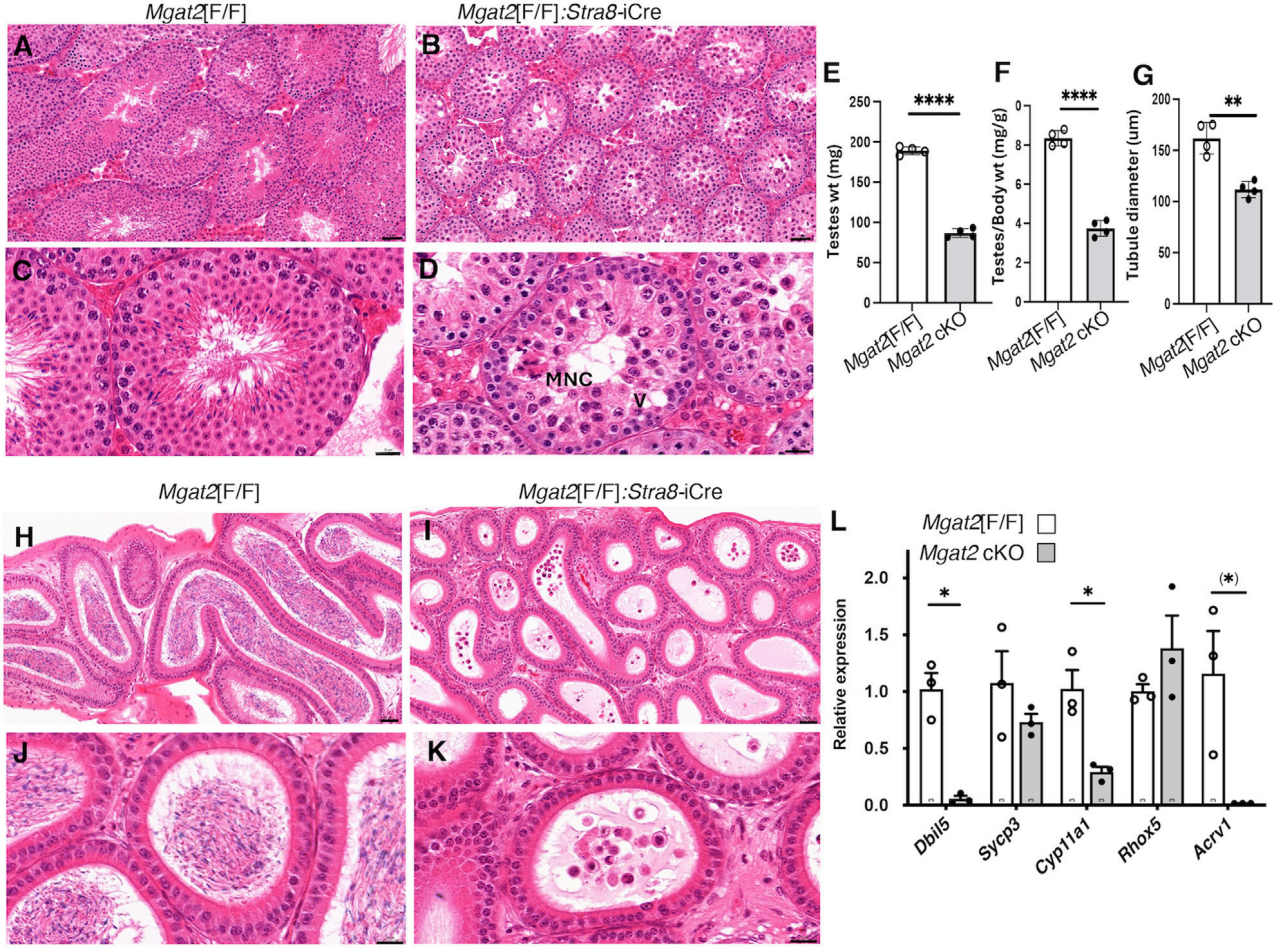
*Mgat2* cKO males do not make sperm. *A*–*D*, representative testis sections from 7-week *Mgat2*[F/F] and *Mgat2*[F/F]:*Stra8*-iCre males stained with H&E (n = 3–4 mice per group, two sections per mouse). An MNC and a vacuole (V) are indicated. Scale bars **(A, B)** 50 μm; **(C, D)** 20 µm. Images from 4-week males are shown in Supplementary Figure S3, **(E)** Weight of both testes in 7-week *Mgat2*[F/F] and *Mgat2* cKO mice. **(F)** Testis to body weight ratio in the same mice. **(G)** Testis tubule diameter in the same mice. Thirty tubules from one section were measured per mouse. **(H–K)** Representative epididymis sections from 7-week *Mgat2*[F/F] and *Mgat2* cKO males stained with H&E (n = 3–4 mice per group, two sections per mouse). Scale bars **(H, I)** 50 μm; **(J, K)** 20 µm. Images of epididymides from 4-week males are shown in Supplementary Figure S3, **(L)** qRT-PCR for marker genes in cDNA of testis cell types from 22-day *Mgat2*[F/F] or *Mgat2* cKO germ cells. Histograms are mean ± SEM. Each symbol represents an individual mouse. Significance was determined by Student’s t-test (two-tailed, unpaired *p < 0.05 **p < 0.01, ****p < 0.001) or one-tailed, unpaired, (*) *p* < 0.05.

Histological examination of testis sections from 7-week *Mgat2*[F/F]:*Stra8*-iCre males showed abnormal morphology compared to *Mgat2*[F/F] testes (Figure 2 and Supplementary Figure S2). Staining by hematoxylin and eosin (H&E) highlighted defects in spermatogenesis in *Mgat2* cKO *versus Mgat2*[F/F] males which had completed the first round of spermatogenesis (Figure 3). It can readily be seen that germ cell numbers were reduced and the types of germ cell within a tubule simplified in *Mgat2*[F/F]:*Stra8*-iCre males (Figures 3A–D). Spermatogonia and spermatocytes (1° and 2°) were present, but round and elongated spermatids were absent and there were no sperm in the lumen of 7-week *Mgat2*[F/F]:*Stra8*-iCre testis tubules. In addition, mutant germ cells were disorganized, and tubules contained one or a few vacuoles and multinuclear cells (MNCs) (Table 1). The latter appear to form from 2° spermatocytes. MNCs were not apparent in testis tubules of 4-week *Mgat2* cKO males when the first wave of spermatogenesis was ∼70% complete (Montoto et al., 2012) (Supplementary Figures S3A, S3B). Compared to 4-week *Mgat2*[F/F] testis, many *Mgat2* cKO tubules were sparsely populated with spermatocytes and lacked round spermatids.

**TABLE 1:** Germ cell defects in 7-week *Mgat2*[F/F]:*Stra8*-iCre testis tubules.

*Mgat2*[F/F] *Stra8*-iCre	MNC <5 nuclei	MNC>5 nuclei	Tubules without MNC	Small vacuoles	Big vacuoles
1	13	3	17	28	5
2	16	5	18	44	8
3	10	4	20	35	3
4	13	5	12	32	11

Testis sections from four 7-week *Mgat2* cKO males were stained with H&E, and germ cell morphologies in 30 round tubules (Tub) per section were scored. *Mgat2*[F/F] sections had no MNC or vacuoles.

**FIGURE 4:**
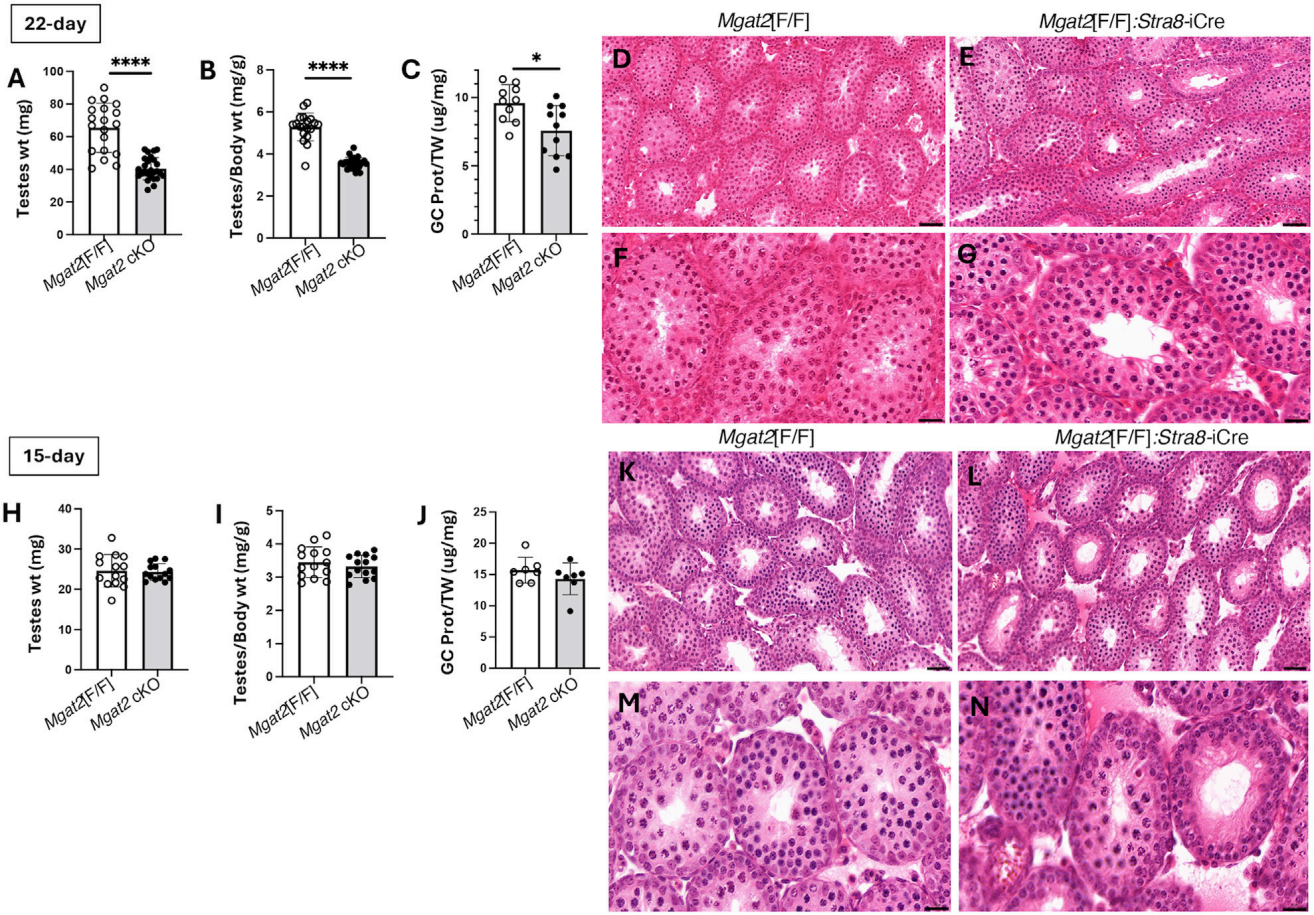
Testis morphology in prepubertal *Mgat2* cKO mice. **(A)** Testes weight in 22-day *Mgat2*[F/F] and *Mgat2* cKO male mice. **(B)** Testes to body weight ratio in the same mice. **(C)** Germ cell (GC) protein to testis weight (TW) ratio in 22-day *Mgat2*[F/F] and *Mgat2* cKO males. **(D, E)** Representative 22-day testis sections stained with H&E. Scale bars 50 µm. **(F, G)** Morphology of single tubules stained with H&E. Scale bars 20 µm. **(H)** Testes weight in 15-day *Mgat2*[F/F] and *Mgat2* cKO males. **(I)** Testes to body weight ratio in the same mice. **(J)** Germ cell protein to testis weight ratio in 15-day *Mgat2*[F/F] and *Mgat2* cKO males. **(K, L)** Representative 15-day testis sections stained with H&E. Scale bars 50 µm. **(M, N)** Morphology of single tubules stained with H&E. Scale bars 20 µm. Histograms are mean ± SEM. Each symbol represents an individual mouse. Significance was determined by Student’s t-test (two-tailed, unpaired); *p < 0.05, ****p < 0.0001.

Testis weight was significantly reduced (Figures 3E, F), and the diameter of testis tubules was smaller (Figure 3G) in 7-week *Mgat2*[F/F]:*Stra8*-iCre males. Consistent with the germ cell defects in 7-week testis tubules of *Mgat2* cKO mice, the epididymides of 7-week mutant mice were devoid of sperm (Figures 3H–K). At 22 days, transcripts specific to round spermatids (*Dbil5*) and elongated spermatids (*Acrv1*) were greatly reduced in the *Mgat2* cKO mice (Figure 3L). Testis weight and germ cell protein content were also decreased in 22-day mutant mice (Figures 4A–C). In addition, testis tubule morphology was abnormal in 22-day *Mgat2*[F/F]:*Stra8*-iCre testes (Figures 4D–G). However, this was not the case for 15-day *Mgat2*[F/F]:*Stra8*-iCre testes. At 15 days, testes weight and protein content of germ cells were similar in *Mgat2*[F/F] and *Mgat2*[F/F]:*Stra8*-iCre males (Figures 4H–J) and the morphology of 15-day *Mgat2*[F/F]:*Stra8*-iCre testis tubules was very similar to controls (Figures 4L–N). In addition, spermatogonia per testis tubule and testis diameter were similar between 15-day *Mgat2*[F/F] and *Mgat2* cKO germ cells (Table 2).

**TABLE 2:** Spermatogonia in 15-day *Mgat2*[F/F] and *Mgat2*[F/F]:*Stra8*-iCre testis tubules.

Mouse genotype	Testis diameter (µm)	Ave. No. Sg	Ave. tubule diameter (µm)
*Mgat2*[F/F]			
1	2115	28.6	99
2	2140	29.5	96
3	2115	30.5	98.5
*Mgat2*[F/F] *Stra8*-iCre			
4	2100	30.0	99
5	2065	27.4	96.8
6	2070	28.0	97.2

Testis sections from 15-day *Mgat2*[F/F] and *Mgat2*[F/F]:*Stra8*-iCre males stained with H&E were scored for testis diameter, number of Sg, and average tubule diameter in 10 round tubules.

### RNA-seq reveals downregulation of genes required for spermiogenesis

Since differences between *Mgat2*[F/F] and *Mgat2*[F/F]:*Stra8*-iCre testis tubules were not apparent at 15 days after birth, this stage was chosen to investigate gene expression changes due to deletion of *Mgat2* in Sg beginning at 3 days after birth (Sadate-Ngatchou et al., 2008). Immuno- and lectin histochemistry showed that 15-day testes from *Mgat2* cKO mice contain germ cells that neither express MGAT2 nor complex N-glycans (Supplementary Figure S4). RNA from germ cells of 4 *Mgat2*[F/F] and 4 *Mgat2*[F/F]:*Stra8*-iCre 15-day males was prepared according to a protocol from Novogene (Supplementary Table S1) and sent for RNA-seq, following polyA enrichment, as described in Experimental procedures.

**FIGURE 5:**
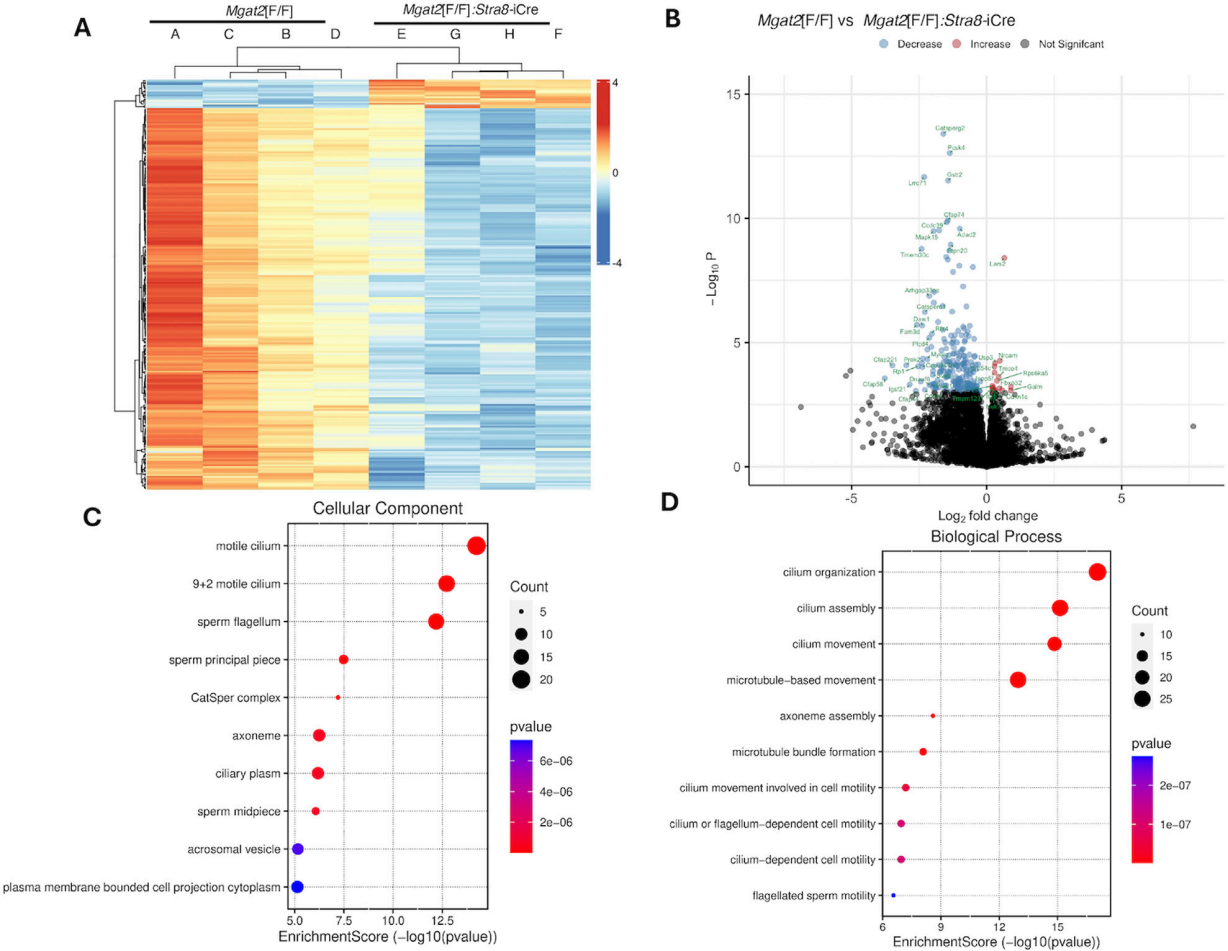
RNA-seq of 15-day *Mgat2*[F/F] and *Mgat2* cKO germ cell RNA. **(A)** Heatmap of protein-coding DEGs at *P*adj < 0.05. **(B)** Volcano plot of the same protein-coding DEGs. **(C)** SRPLOT dot plot of enrichment analysis for GO: Cellular Component. **(D)** SRPLOT dot plot of enrichment analysis for GO: Biological Process.

A Pearson correlation analysis showed high global expression similarities among the eight samples (Supplementary Figure S5). Differential expression analysis identified 302 differentially expressed genes (DEGs) at the false discovery rate (FDR) < 0.05 from a total of 20,187 detected transcripts with the majority of genes downregulated in *Mgat2* 15-day germ cells. The heatmap in Figure 5A compares expression levels of the 245 protein-coding DEGs, confirming that the majority of genes from *Mgat2* cKO germ cells exhibited downregulation. To show the magnitude of expression changes, the data were also presented in a volcano plot, with top DEGs highlighted (Figure 5B). Interestingly, the top downregulated genes are involved in ciliary and flagellar motility and spermiogenesis (the generation of motile, functional spermatozoa), including *Catsperg2*, *Cfap58*, *Cfap221*, and *Cfap44* (Figure 5B). The top 20 protein-coding genes were downregulated by a maximum of ∼14-fold (Supplementary Table S2). The top 10 most significantly downregulated genes are given in Supplementary Table S3. The most upregulated gene with a known function in *Mgat2* cKO germ cells was *Galm* (a galactose mutarotase) at 1.9-fold, followed by *Lars2* (mitochondrial leucyl-tRNA synthetase) at 1.6-fold (Figure 5B). Overall, there were only 17 upregulated protein-coding genes.

*Mgat2*[F/F] *versus Mgat2*[F/F]:*Stra8*-iCre DEGs were examined by gene ontology (GO) pathway enrichment analysis. The top 245 protein-coding DEGs were compared by overrepresentation analysis and pathways plotted using SRPLOT (Tang et al., 2023). The top cellular component GO terms affected by the removal of *Mgat2* were motile cilium, sperm flagellum, and the CatSper complex, the cation channel of sperm necessary for sperm motility (Figure 5C). The top biological process GO terms affected in *Mgat2* cKO germ cells were cilium assembly, organization, and movement (Figure 5D). Thus, the most downregulated pathways in *Mgat2* cKO germ cells at 15 days after birth are involved in spermiogenesis, a process which pertains to the formation of sperm that begin to appear in the mouse testis lumen ∼40 days after birth (Montoto et al., 2012).

**FIGURE 6:**
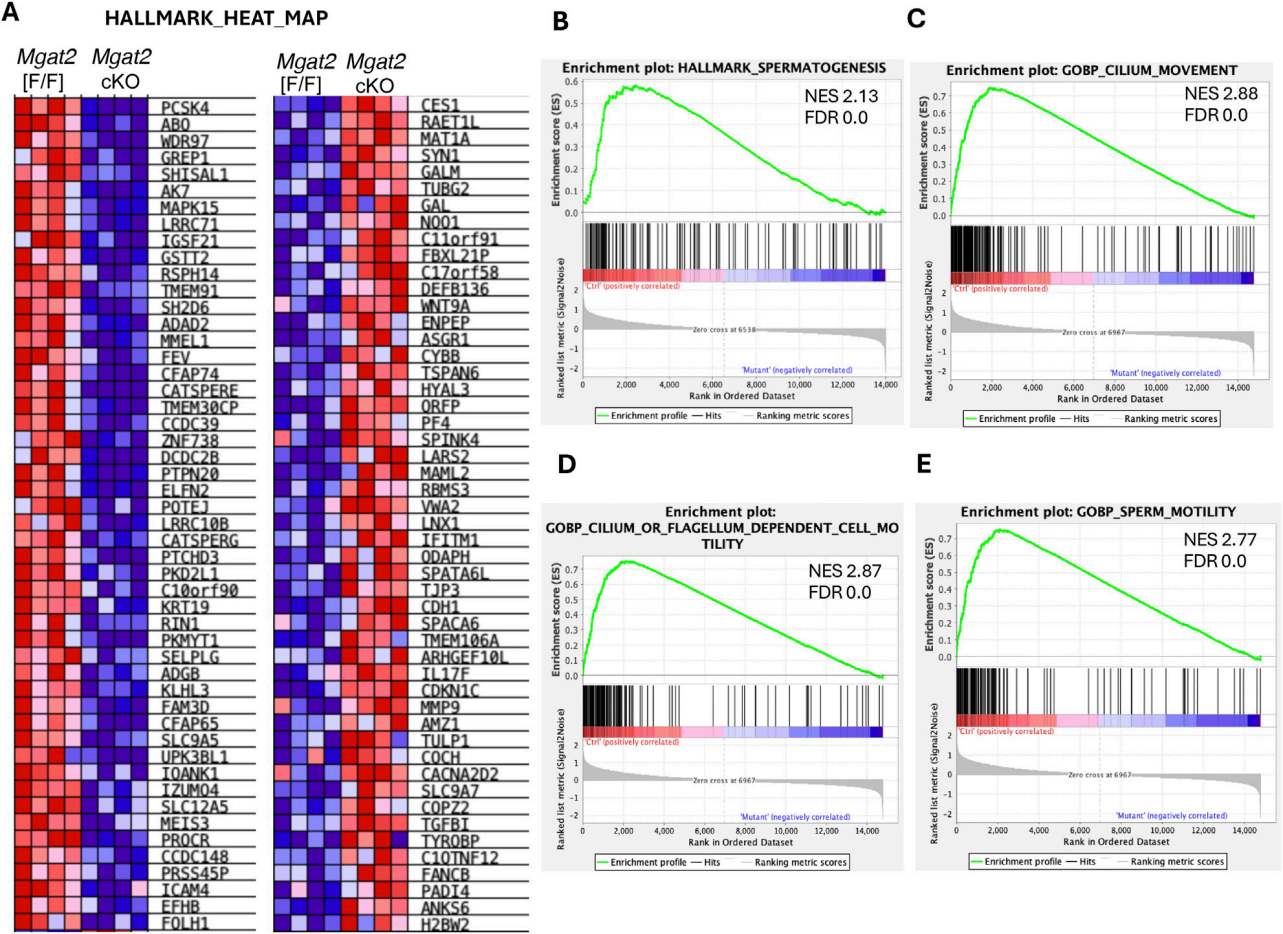
GSEA of protein-coding DEGs. **(A)** Heatmap of up- and downregulated genes in 15-day *Mgat2*[F/F] and *Mgat2* cKO germ cells from the mouse HALLMARK collection of gene sets. **(B)** Top gene set determined by GSEA in the HALLMARK collection. **(C–E)** Top three gene sets determined by GSEA of the Gene Ontology biological process (GOBP) mouse c5 collection.

We also performed gene set enrichment analysis (GSEA) to identify mouse gene sets significantly enriched in the up- and downregulated genes between *Mgat2*[F/F] and *Mgat2* cKO germ cells, using the HALLMARK GSEA collection (Figure 6A). The most significant gene set is HALLMARK_SPERMATOGENESIS with a normalized enrichment score (NES) of 2.13 (Figure 6B). Other gene sets of high significance were found in the mouse GOBP c5 collection and are related to cilium, flagellar, and sperm movement (Figures 6C–E).

To gain insights into potential signaling pathways affected in *Mgat2* cKO germ cells, we performed ingenuity pathway analysis (IPA) using the 245 protein-coding DEGs. Downstream effects analysis in the category organismal injury and abnormalities predicted an increase in sperm disorder (Z-score 2.96, overlap *p*-value 4.2E-05), infertility condition (Z-score 2.00, overlap *p*-value 5.04E-05), and azoospermia or oligospermia (Z-score 3.00, overlap *p*-value 1.07E-02). In the cell death and survival category, apoptosis of male germ cells was predicted to increase (Z-score 2.00, overlap *p*-value 3.76E-02).

**FIGURE 7:**
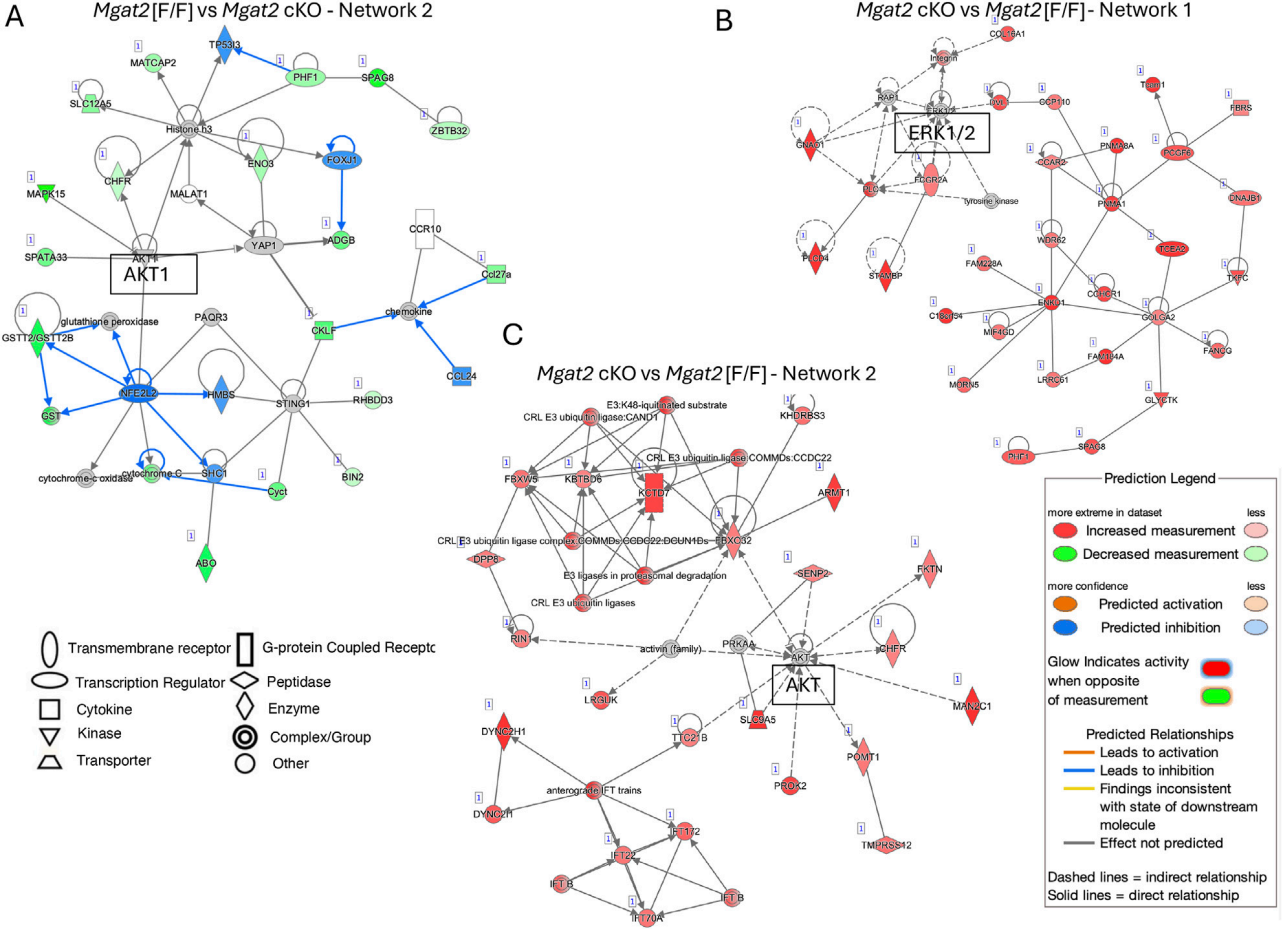
Pathway enrichment analysis by IPA. **(A)** Network 2 in the IPA analysis of 20,167 mapped IDs. *Mgat2*[F/F] vs. *Mgat2* cKO germ cell DEGs using Expr log ratio, Expr fold-change, Expr *p* < 0.05, and *P*adj < 0.05. **(B)** Network 1 in the IPA analysis of 20,167 mapped IDs. *Mgat2*[F/F] vs. *Mgat2* cKO germ cell DEGs using only *P*adj < 0.05. **(C)** Network 2 for the IPA analysis described in **(B)**.

To map biological relationships of *Mgat2* DEGs to the published information in IPA, network analysis was performed. From an analysis using four parameters (log ratio, log_2_fc, *p*-value < 0.05, and *P*adj value < 0.05), Network 2 with 35 molecules, a score of 30 and 17 focus molecules predicted AKT1 to have increased activity (Figure 7A, Supplementary Table S4). When only *P*adj < 0.05 was used as a parameter, Network 1 with a score of 65 and 30 focus molecules identified ERK1/2 as a focal point with no prediction of activity (Figure 7B, Supplementary Table S5); Network 2 with a score of 45 and 23 focus molecules identified a central role for AKT with no prediction of up or down activity (Figure 7C, Supplementary Table S6).

**FIGURE 8:**
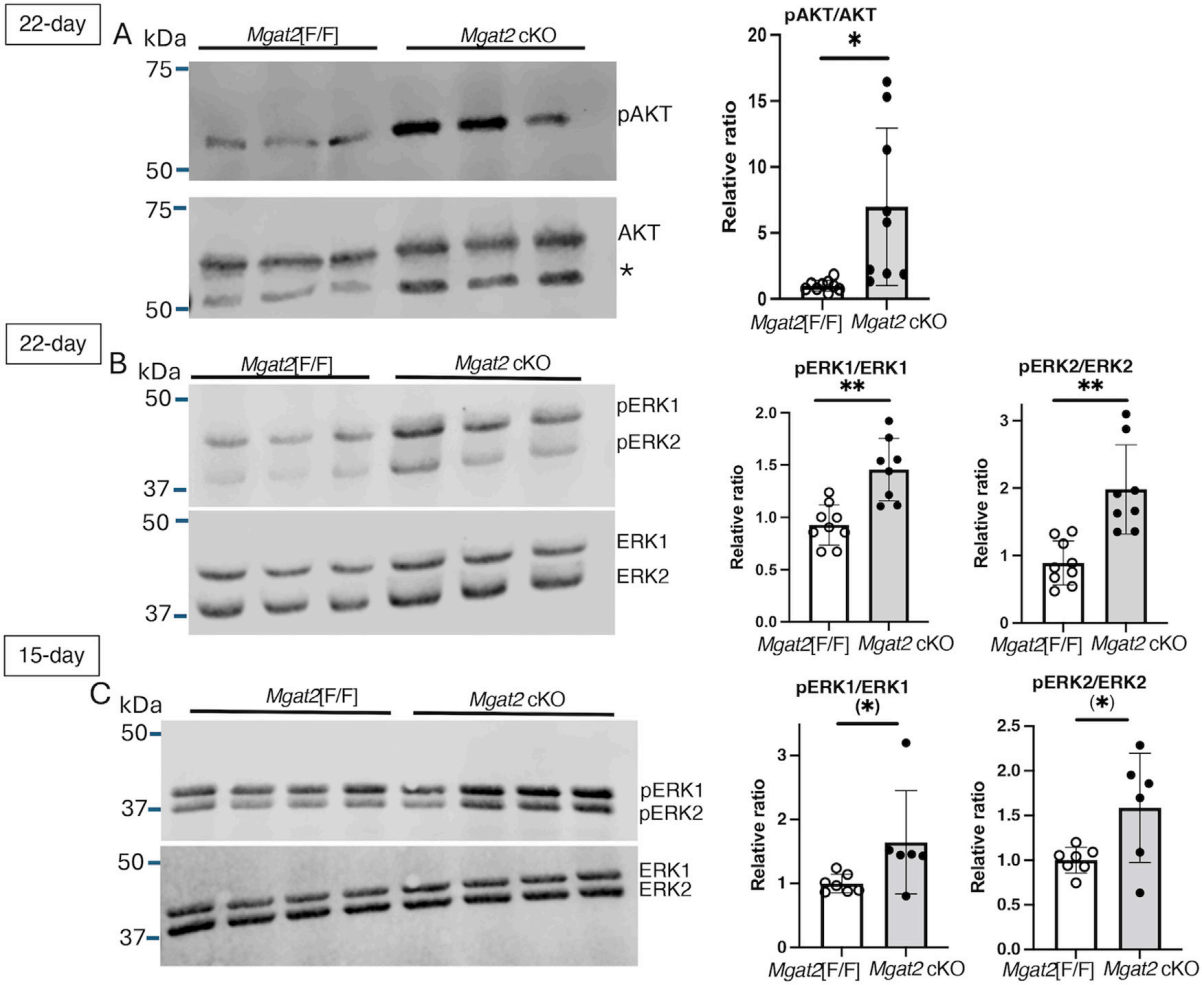
AKT and ERK signaling in *Mgat2* cKO germ cells. Germ cell lysates were prepared from 22-day or 15-day control and *Mgat2* cKO males. **(A)** Western blots of pAKT and AKT in 22-day *Mgat2*[F/F] or *Mgat2* cKO germ cell lysates. The star designates a non-specific band consistently detected with the Ab to AKT. Histograms at right show the relative ratio of pAKT:AKT in independent lysates. **(B)** Western blots of phosphorylated and total ERK1 and ERK2 in *Mgat2*[F/F] or *Mgat2* cKO germ cell lysates and histograms showing the relative ratio of pERK1/2:ERK1/2 in 22-day germ cells. **(C)** Western blots of phosphorylated and total ERK1 and ERK2 in *Mgat2*[F/F] or *Mgat2* cKO germ cell lysates and histograms showing the relative ratio of pERK1/2:ERK1/2 in 15-day germ cells. Histograms are mean ± SEM. Each symbol represents the lysate from an individual male. Significance was determined by Student’s t-test (two-tailed, unpaired), *p < 0.05, **p < 0.01 or one-tailed, unpaired *t*-test (*) *p* < 0.05.

To validate predicted changes of AKT and ERK1/2 from our RNA-seq results, we analyzed AKT and ERK1/2 signaling in 22-day and 15-day germ cell lysates by Western analysis. Levels of pAKT in relation to total AKT were significantly increased, revealing activation of AKT signaling in 22-day *Mgat2* cKO germ cells (Figure 8A and Supplementary Figure S6). Similarly, activation of ERK1 and ERK2 signaling was observed in the same *Mgat2* cKO germ cell extracts at both 22 days and also at 15 days after birth (Figures 8B, C, Supplementary Figures S7, S8). Therefore, loss of MGAT2 in developing male germ cells caused an activation of both AKT and ERK signaling.

## Discussion

Roles for glycans in mammalian spermatogenesis have been identified in many engineered mouse models (Fukuda and Akama, 2003b; Akintayo and Stanley, 2019). However, a large number of “testis-specific” genes, so termed because they are primarily or only expressed in testis, are not essential for spermatogenesis in the mouse (Miyata et al., 2016; Akintayo et al., 2020). The first mouse lacking complex N-glycans in male germ cells carried a global deletion of *Man2a2* (Figure 1B) (Fukuda and Akama, 2002; Fukuda and Akama, 2003a). *Man2a2*-null males are produced in low numbers and are infertile. Testis size and germ cell numbers are reduced; germ cells are widely separated and prematurely released from Sertoli cells, and MNCs form between germ cells. The second mouse reported to lack complex N-glycans was a conditional knockout of *Mgat1* in spermatogonia (Batista et al., 2012). Germ cells conditionally null for *Mgat1* appear morphologically normal, but spermatids fuse to form MNC. *Mgat1*[−/−] germ cells at 22 days have reduced ERK1/2 signaling (Biswas et al., 2018; Biswas et al., 2023). In the case of *Mgat2* null germ cells reported here, the spermatogenic phenotype was more similar to males lacking MAN2A2 in having reduced testis size and germ cell numbers. By contrast, however, few MNCs were formed per tubule and germ cells were not widely separated in *Mgat2* cKO males. Thus, the defective spermatogenesis phenotype differed in germ cells lacking MGAT2 *versus* those lacking MAN2A2 or MGAT1. For this reason, the mechanistic basis of their respective defects could not be ascribed solely to a unified mechanism related to a lack of complex N-glycans on germ cell glycoproteins. It seems more likely that the different immature N-glycans accumulated in each model lead to differently dysregulated signaling in germ cells—inhibition (MGAT1) *versus* activation (MGAT2) of growth factor receptor signaling in male germ cells. Substantial experimental work would be required to determine the molecular origins of the *Mgat2* null germ cell phenotype, including the molecular basis of MNC formation. We speculate that the different hybrid N-glycans generated in the respective mutant germ cells may be responsible for the different spermatogenic defects observed and that MNC formation may be a stochastic event.

MGAT2 transfers GlcNAc to the GlcNAc_1_Man_3_GlcNAc_2_Asn hybrid N-glycan generated by the actions of MGAT1 and MAN2A2 to form biantennary N-glycan GlcNAc_2_Man_3_GlcNAc_2_Asn (Figure 1B). If MGAT2 is absent, the single GlcNAc added by MGAT1 may be extended by Gal and GlcNAc transferases to form pLN, and this has been found to occur in mutant cells and tissues (Wang et al., 2001; Mortales et al., 2020a; Mortales et al., 2020b). The consequence of extension with pLN is to partially, or almost completely, rescue phenotypic effects of deleting *Mgat2*. For example, in *Mgat2* cKO T (Mkhikian et al., 2016) or B cells (Mortales et al., 2020a), the hybrid N-glycan generated is extended to form pLN (detected by L-PHA binding), resulting in developmental defects being greatly ameliorated. However, due to the absence of L-PHA binding by *Mgat2* cKO germ cells and the severe effects of the loss of MGAT2 in germ cells, it can be concluded that pLN was not generated in *Mgat2* cKO germ cells, despite the absence of N-glycan structural data. In addition, transcripts for the glycosyltransferases that can generate pLN were not significantly increased in *Mgat2* cKO germ cells (see Figure 5 and RNA-seq data). Finally, and strikingly, the phenotype of *Mgat2* cKO germ cells was quite different from germ cells lacking MGAT1 or MAN2A2. Loss of MGAT2 gave a more severe inhibition of spermatogenesis that arose earlier in spermatogenesis before the appearance of spermatids. It also involved reduced germ cell numbers and markedly reduced the testis tubule diameter. Thus, we hypothesize that the truncated hybrid N-glycan formed on glycoproteins in *Mgat2* cKO germ cells provides the basis of inhibited spermatogenesis. Similarly, the blocked spermatogenesis in *Mgat1* cKO germ cells and *Man2a2* KO mice might arise from the respective immature N-glycan that accumulates on glycoproteins in those mutant germ cells. Notably, the accumulation of the Man_5_GlcNAc_2_Asn substrate of MGAT1 correlates with reduced ERK1/2 signaling in 22-day germ cells, and this is accompanied by reduced *Pdgfra* transcripts (Biswas et al., 2018; 2023). By contrast, the accumulation of the GlcNAc_1_Man_3_GlcNAc_2_Asn substrate of MGAT2 was accompanied by an increase in ERK1/2 signaling and an increase in AKT signaling (Figure 8). This upregulation of signaling pathways that normally leads to cell proliferation may be a stress response of germ cells lacking MGAT2, which are predicted to be progressing toward apoptosis. Importantly, no significant change in expression of a growth factor receptor was observed amongst the DEGs, indicating no particular avenue to pursue to discover a mechanistic basis for upregulated ERK and AKT signaling in *Mgat2* cKO germ cells. Nevertheless, MGAT2 might prove to be a good candidate for inhibition by a small molecule that could lead to the development of a male contraceptive.

## Experimental procedures

### Mice

*Mgat2*[F/F] mice (JAX ^®^ Strain 006892, B6.129-Mgat2tm1Jxm/J) were a kind gift of Michael Demetriou (UC Irvine) with express permission from the Jackson Laboratories (Bar Harbor, Maine). Their development was previously described (Mkhikian et al., 2016). The *Mgat2*[F/F] mice were crossed to mice carrying one copy of the *Stra8*-iCre transgene (B6.FVB-Tg(Stra8-icre)1Reb/LguJ; JAX strain: 017490; (Sadate-Ngatchou et al., 2008)) purchased from the Jackson Laboratories and backcrossed for >10 generations to C57BL6/J mice. The resulting *Mgat2*[F/F] control and *Mgat2*[F/F]:*Stra8*-iCre experimental mice were black and were bred by crossing *Mgat2*[F/F]:Stra8-iCre females with *Mgat2*[F/F] males. The mice were housed in a barrier facility and allowed to eat chow 5,058 (LabDiet, Richmond, IN) and drink water *ad libitum*. Pups were genotyped by PCR of tail DNA, as described in Figure 1C and Supplementary Figure S1B using primers given in Supplementary Table S7. At various ages after birth, mice were sacrificed by asphyxiation in a CO_2_ chamber, followed by cervical dislocation. Mice were weighed, testes removed, weighed, and used for experiments, as described below. Mouse experiments were performed following the approval by the Albert Einstein IACUC under protocol numbers 20170709 and 00001311.

### Histological analysis

Testes were fixed in Bouin’s fixative (cat # 100503-962, Electron Microscopic Sciences, Radnor, PA) for a minimum of 48 h at room temperature (RT) before processing and paraffin embedding at the Albert Einstein Histology and Comparative Pathology Core. Serial sections of 5–6 μm were arrayed on slides with a positive charge and stained with H&E or used for histochemistry. Images of testis sections were obtained using a Perkin Elmer P250 high-capacity slide scanner by the Analytical Imaging Facility at the Albert Einstein College of Medicine. Image analysis was performed using proprietary software from CaseViewer (3DHISTECH P250 high-capacity slide scanner, Perkin Elmer, Waltham, MA). Scale bars are embedded and can be viewed by enlarging the image. Redrawn scale bars are shown in figures.

### Immunohistochemistry

Immunohistochemistry was performed following the protocol for paraffin-embedded sections from Abcam (https://www.abcam.com/protocols). Histo-Clear reagent (cat. # HS-200, National Diagnostics, Atlanta, GA) was used to deparaffinize sections, and rehydration was performed using Decon Labs ethanol (BioPharmWorld, Frederick, MD) by washing twice with 100% ethanol for 3 min, followed by 95%, 80%, and 70% ethanol for 3 min each. To quench endogenous peroxidase, slides were exposed to 3% H_2_O_2_ (cat #H1009, Sigma-Aldrich, St. Louis, MO) in phosphate buffered saline with 1 mM CaCl_2_ and 1 mM MgCl_2_, pH 7.5 (PBS) for 20 min, followed by epitope retrieval in boiling citrate buffer (pH 6.0, Diagnostic BioSystems, Pleasanton, CA) for 20 min, and a subsequent 20-min incubation at RT in fresh citrate buffer. Sections were then incubated in PBS for 5 min at RT and blocked for 1 h at RT in 5% goat serum in PBS blocking buffer (cat # 927-700001, LICORbio™, Lincoln, NE). The primary antibody (1° Ab), unless otherwise specified, was diluted 1:100 in PBS blocking buffer and incubated overnight at 4°C. After rinsing twice with PBS, slides were incubated with the biotinylated secondary antibody diluted in PBS blocking buffer (LICORbio™) for 1 h at RT. Following two PBS washes, peroxidase substrate 3, 3′-diaminobenzidine (DAB; Cat# SK-4100, Vector laboratories, Burlingame, CA) was applied following the manufacturer’s protocol. Tissues were counterstained with the hematoxylin solution (cat # Ref 1.05174.0500, Sigma-Aldrich) diluted at 1:4 ratio with double-distilled water, rinsed in tap water, dipped twice in acid ethanol (0.3% HCl in 100% ethanol), rinsed in tap water, dipped in 2.5% ammonium hydroxide solution diluted from 29.5% ammonium hydroxide (cat# A-6899, Sigma-Aldrich), rinsed in tap water, dehydrated by washing twice with 70% ethanol followed by 95% ethanol, and then 100% ethanol for 3 min followed by Histo-Clear (National Diagnostics). Sections were mounted using Permount^®^ reagent (cat# SP15–100, Fisher Scientific, Fair Lawn, NJ). Images of testis sections were generated using the 3DHISTECH Panoramic 250 Flash II slide scanner by the AIF at the Albert Einstein College of Medicine.

### Lectin histochemistry

After dewaxing and rehydrating tissue sections as described above, sections were immersed in 0.5% H_2_O_2_ and 0.4% HCl in absolute methanol for 30 min at RT to inactivate peroxidase, followed by two washes in double-distilled water and two washes in Tris-buffered saline, pH 7.5 (TBS). Subsequently, the sections were incubated for 5 min in 20 mg/mL proteinase K (cat # AM2546, Invitrogen) in TBS at RT in a humidified chamber. After two washes in TBS, the sections were incubated for 1 h at RT in 10 μg/mL biotinylated plant lectin (L-PHA or GSA-II, Vector Laboratories) in TBS. Following three washes in TBS, the sections were incubated in 5 μg/mL streptavidin peroxidase for 1 h at RT. After three washes in TBS, the DAB reagent (Vector Laboratories) was used for development until control samples were clearly stained. The reaction was stopped in distilled water, and the sections counterstained with hematoxylin, followed by dehydration, clearing, and mounting prior to imaging by the AIF, as described above.

### Germ cell isolation

After dissection, testes were weighed and collected in DMEM:F12 (cat # 11330-032, Gibco, Grand Island, NY) on ice, tunica albuginea were removed, and germ cells were isolated according to Chang et al. (2011) with some modifications. For 22-day mice, tubules were transferred to 10 mL of enzyme solution I (0.5 mg/mL collagenase Type I (cat #C0130-1G, Sigma-Aldrich) and 200 μg/mL DNase I (cat # DN25-100, Sigma-Aldrich) in DMEM:F12 medium). After a brief vortex, the mixture was incubated for 40 min at 33°C in a shaking water bath (100 oscillations per min with manual shaking every 10 min). Following dispersion, tubules were allowed to settle, and the supernatant was discarded. After a wash in 10 mL DMEM:F12 at RT, tubules were resuspended in 10 mL DMEM:F12 and layered onto 40 mL of 5% Percoll (cat # 17-0891-02, GE Healthcare Bio-sciences AB, Uppsala, Sweden) in HBSS (cat # 55-022-PB, Mediatech Inc., Manassas, VA) and left to settle for 20 min at RT. After discarding the top 45 mL, the remaining 5 mL was transferred to a new tube containing 10 mL of enzyme solution II (200 μg/mL DNase I and 1.25 mg/mL trypsin from Sigma-Aldrich in DMEM:F12) and incubated at 33°C in a shaking water bath for 40 min, as described above. Following tissue dissociation, 3 mL of charcoal-stripped fetal bovine serum (FBS, cat # A33821-01, Gibco) was added, and the cells were pipetted 20 times with a 10-mL pipette to dissociate clumps. The suspension was filtered through a 70-µm strainer followed by a 40-µm nylon cell strainer (cat # 352350 and 352340, respectively; Falcon Corning Inc, Corning, NY) and centrifuged at 800 *g* for 10 min at 4°C. After resuspending the cell pellet in 1 mL of cold HBSS (cat # 14175-095, Gibco), cells were counted using a hemocytometer. They were centrifuged at 800 *g* for 10 min at 4°C and stored as a pellet at −80°C.

For 15-day germ cells, the method of Dean et al. (Wang et al., 2019) with slight modifications was used. After removal of tunica albuginea, testis tubules were placed in 5 mL of enzymatic solution 1, followed by gentle agitation in a shaking water bath at 37°C for 20 min (75 oscillations per min with manual shaking every 6 min). Tubules were then permitted to settle, and the supernatant was removed. After a wash with 5 mL of fresh DMEM:F12, tubules were resuspended in 5 mL fresh DMEM:F12 and transferred to a new tube containing 5 mL of enzyme solution II. After 20 min gentle agitation in a 37°C shaking water bath at 75 oscillations per min with manual inversion every 5 min, 20% FBS (cat # A42C22A, GeminiBio, West Sacramento, CA) was added, and clumps were dissociated by resuspending the cells 20 times using a 5-mL pipette. The suspension was filtered through a 70-µm filter (pre-wet with 1 mL DMEM:F12, followed by centrifugation at 1000 *g* for 10 min at 4°C. The cell pellet was resuspended in 1 mL of cold HBSS (Gibco) and counted using a hemocytometer. After centrifugation at 800 *g* for 10 min at 4°C, the germ cell pellet was stored at −80°C.

### Western blot analysis

Testis lysates were prepared using RIPA Lysis Buffer (cat # 20-188, Millipore, Temecula, CA), following the Abcam tissue lysate preparation protocol. In brief, one frozen testis was homogenized in 200 μL RIPA buffer containing 0.1% SDS, protease inhibitor cocktail (cat # 05892791001, Roche Diagnostics GmbH, Mannheim, Germany), and phosphatase inhibitor (cat # 04906845001, Roche Diagnostics). The lysate was continuously agitated using a pellet pestle (cat # 749521-1590, Kimble, Millville, NJ) for 5 min, incubated for 30 min on ice, and centrifuged at 12,000 rpm for 10 min at 4°C. The supernatant was transferred to a fresh tube, and protein was determined using the Bradford-based colorimetric assay (cat # 500-0006, Bio-Rad Protein assay, Bio-Rad, Hercules, CA). The lysate was frozen at −80°C.

Frozen germ cells were lysed in 75 μL buffer (1% IGEPAL (cat #I3021, Sigma-Aldrich), 1% TX-100 (cat #T9284, Sigma-Aldrich), 0.5% deoxycholate (cat #D6750, Sigma-Aldrich), 1.5× phosphatase inhibitor (Roche), and Roche protease inhibitor cocktail dissolved in double-distilled water. The lysate was incubated for 30 min on ice, followed by centrifugation for 5 min at 5,000 g at 4°C. The supernatant was transferred to a fresh tube, and protein quantified using the Bradford-based colorimetric assay. Germ cell lysate was stored at −80°C.

For the detection of MGAT2, 60 μg of protein was separated by 10% SDS-PAGE. Following transfer to a polyvinylidene fluoride (PVDF) membrane in transfer buffer containing 10% methanol for 90 min, the membrane was blocked in TBS Odyssey blocking buffer (TOBB, cat #P/N 927-600001, LICORbio™) for 1 h at 37°C and incubated overnight at 4°C with anti-MGAT2 primary Ab (cat # LS-C40495, LS Bio, Lynnwood, WA) diluted 1:400 in TBS blocking buffer. Secondary Ab, which was donkey anti-rabbit IRDye^®^ 800CW (LICORbio™) at a dilution of 1:8000 in TBS blocking buffer, was applied for 1 h at RT, followed by four washes with TBST (TBS and with 0.05% Tween 20 (cat #P7949, Sigma-Aldrich). Imaging was performed using a LI-COR Odyssey scanner.

For the detection of AKT and ERK, 60 μg of the germ cell extract was electrophoresed by 10% SDS-PAGE and transferred to a polyvinylidene fluoride (PVDF) membrane. The membrane was blocked for 2 h at RT using TOBB, followed by overnight incubation at 4°C with primary Ab in TOBB. Antibodies obtained from cell signaling technology (Danvers, MA) were the following: rabbit monoclonal antibody (mAb) D13.14.4E targeting pERK1/2 (cat # 4370), mouse mAb 3A7 targeting ERK1/2 (cat # 9107), mouse mAb 40D4 targeting AKT (cat # 2920), and rabbit mAb D9E targeting pAKT (cat # 4060). The secondary Abs used were donkey anti-rabbit IgG IRDye 800CW (cat #P/N926-32213, LI-COR) and goat anti-mouse IgG DyLight™ 680 (cat # 35519, Invitrogen). After incubation, membranes were rinsed four times in TBST and incubated in LI-COR secondary Abs in TOBB for 1 h at RT in the dark. Subsequent washes were four times with TBST and twice with TBS. Imaging was performed using the LI-COR Odyssey Fc imaging system, and band intensity determination was obtained using LI-COR^®^ Acquisition Software (LICORbio™).

### Nuclei acid isolation

Total RNA was isolated from frozen testis using TRIzol (cat # 15596018, Invitrogen) and the manufacturer’s protocol. TRIzol was also used to extract genomic DNA from frozen germ cells and total RNA from germ cells of 22-day males, according to the manufacturer’s protocol. To obtain total RNA from 15-day frozen germ cells, RNAZol (cat #R4533, Sigma-Aldrich) was used with the recommended protocol for RNA extraction. In brief, for both RNA extraction methods, 5 × 10e6 frozen germ cells were homogenized using the Kimble pellet pestle in and 1 mL TRIZol or RNAZol, followed by a 5 min incubation at RT. Subsequently, 200 µL of chloroform was added, the tubes vortexed for 15 s, left at RT for 2–3 min, and then centrifuged at 12,000 g for 15 min at 4°C. The aqueous phase was carefully transferred to a new tube, and 500 µL of isopropanol was added. After incubating for 10 min in ice, the samples were centrifuged at 12,000 g for 10 min at 4°C. The RNA pellet was washed once with 1 mL 70% ethanol, vortexed briefly, and centrifuged at 7,500 g for 5 min at 4°C. The RNA pellet was air-dried for 5–10 min and dissolved in 30–50 µL of RNase-free water (cat #H20MB0106, Millipore Corp., Billerica, MA). The samples were placed in a 55°C–60°C water bath for 15–20 min, and the RNA concentration was measured using a NanoDrop ND1000 spectrophotometer. RNA quality (RQN) was determined by the Albert Einstein Genomics Core Facility using Bioanalyzer software on a Pico RNA Chip type.

### Quantitative RT-PCR

The synthesis of cDNA was performed with 2 µg total RNA and the ReverTra Ace^®^ qPCR RT Master Mix with gDNA Remover (cat # FSQ-301, Dc. DiagnoCine Toyobo, Osaka, Japan) in accordance with the manufacturer’s guidelines. Quantitative RT-PCR (qRT-PCR) was performed in triplicate in a 384-well dish in a ViiA 7 Real Time PCR System (Thermo Fischer Scientific, Waltham, MA). The assessment of gene expression was performed relative to *Gapdh* and *Actb* using the ddCT method. The primers used for both RT-PCR and qRT-PCR analyses are listed in Supplementary Table S7.

### RNA-seq analysis

Total RNA from 15-day germ cells of *Mgat2*[F/F] and *Mgat2*[F/F]:*Stra8*-iCre testes was provided at 3 ng/μL to the Genomics Core Facility to obtain RQN values. All samples with an RQN of 10.0. RNA at ∼25 ng/μL in RNAse-free water in 1.5 mL microtubes were sent on dry ice to Novogene (Sacramento, CA) for library preparation and RNA-seq. Non-stranded mRNA-Seq libraries (polyA enrichment) were prepared by Novogene for 150 base pair paired-end (PE) sequencing on an Illumina HiSeq platform (NovaSeq Illumina PE150 with 6 G raw data per sample). After quality controls of the reads, STAR (v2.7.9a) (Dobin et al., 2013) was used to align the PE reads to the mouse reference genome (mm39). RSEM (v1.3.3) (Li and Dewey, 2011) was used to compute read counts, fragments per million fragments mapped (FPKM), and transcripts per million (TPM) using gene annotations from the Ensembl database (v105). Genes with a TPM≥1 in at least one of the eight samples were retained for downstream analyses. DEseq2 (v1.38.3) (Love et al., 2014) was applied to perform differential expression analysis. A false discovery rate (FDR) < 0.05 was used to identify DEGs. Protein-coding DEGs are given in Supplementary Table 1.

### Bioinformatics

Pathway enrichment analysis was performed with overrepresentation analysis using the DEGs and GSEA. We acknowledge the use of GSEA software and the Molecular Signature Database (MSigDB) (https://www.gsea-msigdb.org/gsea/index.jsp) (Subramanian et al., 2005) using all protein-coding genes or genes ranked by their expression difference between *Mgat2* cKO germ cells and controls. The IPA (RRID:SCR_008653) algorithm by QIAGEN, Inc. (https://www.qiagenbioinformatics.com/products/ingenuity-pathway-analysis) (Kramer et al., 2014), was utilized to perform pathway analysis of protein-coding gene DEGs with a fold-change of ±1.5 and an overlap *p*-value below 0.05. Gene Ontology enrichment analyses were plotted by SRPLOT (https://www.bioinformatics.com.cn), (Tang et al., 2023), an online platform for data analysis and visualization.

### Statistical analysis

Bar graphs comparing samples from individual *Mgat2*[F/F] and *Mgat2* cKO mice show mean ± SEM reflecting the variation between animals. Calculation of *p*-values was performed using the unpaired, two-tailed Student’s t-test or one-way ANOVA with GraphPad Prism 8.0 (GraphPad Software Inc., La Jolla, CA), unless otherwise noted. Statistical significance is indicated by *p < 0.05, **p < 0.01, ***p < 0.001, and ****p < 0.0001.

## Data Availability

The datasets presented in this study can be found in online repositories. The names of the repository/repositories and accession number(s) can be found at: https://www.ncbi.nlm.nih.gov/, GSE264025.

## Abbreviations

cKO, conditional knockout; KO, global knockout; PBS, phosphate-buffered saline with cations; TBS, Tris-buffered saline; MNC, multinuclear cell; H&E, hematoxylin and eosin; Sg, spermatogonia; Sc, spermatocyte; RSt, round spermatid; ESt, elongated spermatid; Sp, sperm; GC, germ cell; TW, testis weight; L-PHA, *P. vulgaris* leukoagglutinin; GSA-II, *G. simplicifolia* II; gDNA, genomic DNA; qRT-PCR, quantitative reverse-transcriptase PCR; 1° Ab, primary antibody; mAb, monoclonal antibody; DEGs, differentially expressed genes; FDR, false discovery rate; GO, Gene Ontology; IPA, ingenuity pathway analysis; GSEA, gene set analysis; NES, normalized enrichment score.
