# Supplementary material for: Deletion of Mgat2 in spermatogonia blocks spermatogenesis
Source: Front Cell Dev Biol. 2024 Sep 19;12:1428715. doi: 10.3389/fcell.2024.1428715 (PMC11447316; doi:10.3389/fcell.2024.1428715)
Supplement: Supplementary file 1 [file DataSheet1.pdf]

Supplemental Information

Deletion of *Mgat2* in Spermatogonia Blocks Spermatogenesis

Mohd Shamoon Asmat[1], Xiang Yu Zheng[2], Mohd Nauman[1], Deyou Zheng[2, 3], Pamela Stanley[1, *]

[1] Department of Cell Biology, [2] Genetics, [3] Neurology and Neuroscience, Albert Einstein College of Medicine, New York, NY, 10461.

*Corresponding author: pamela.stanley@einsteinmed.edu

Supporting Figures S1 to S8

Figure S1. Germ cells enrichment, gDNA genotyping and western analysis.

Figure S2. *Mgat2* cKO germ cells do not express MGAT2 or complex N-glycans.

Figure S3. *Mgat2* cKO testis tubules at 4 weeks.

Figure S4. *Mgat2* cKO germ cells do not express MGAT2 or complex N-glycans at 15 days.

Figure S5. Pearson correlation coefficient for RNA-seq samples.

Figure S6. AKT signaling in 22-day *Mgat2*[F/F] versus cKO germ cells.

Figure S7. ERK signaling in 22-day *Mgat2*[F/F] versus cKO germ cells

Figure S8. ERK signaling in 15-day *Mgat2*[F/F] versus cKO germ cells.

Supporting Tables S1 to S7

Table S1. RNA quality for RNA-seq.

Table S2. Top down-regulated genes in *Mgat2* cKO germ cells.

Table S3. Most significantly down-regulated genes in *Mgat2* cKO germ cells.

Table S4. Molecules in IPA Network 2 in Fig.7*A*.

Table S5. Molecules in IPA Network 1 in Fig. 7*B*.

Table S6. Molecules in IPA Network 2 in Fig. 7*C*.

Table S7. Primers used in qRT-PCR and genotyping.

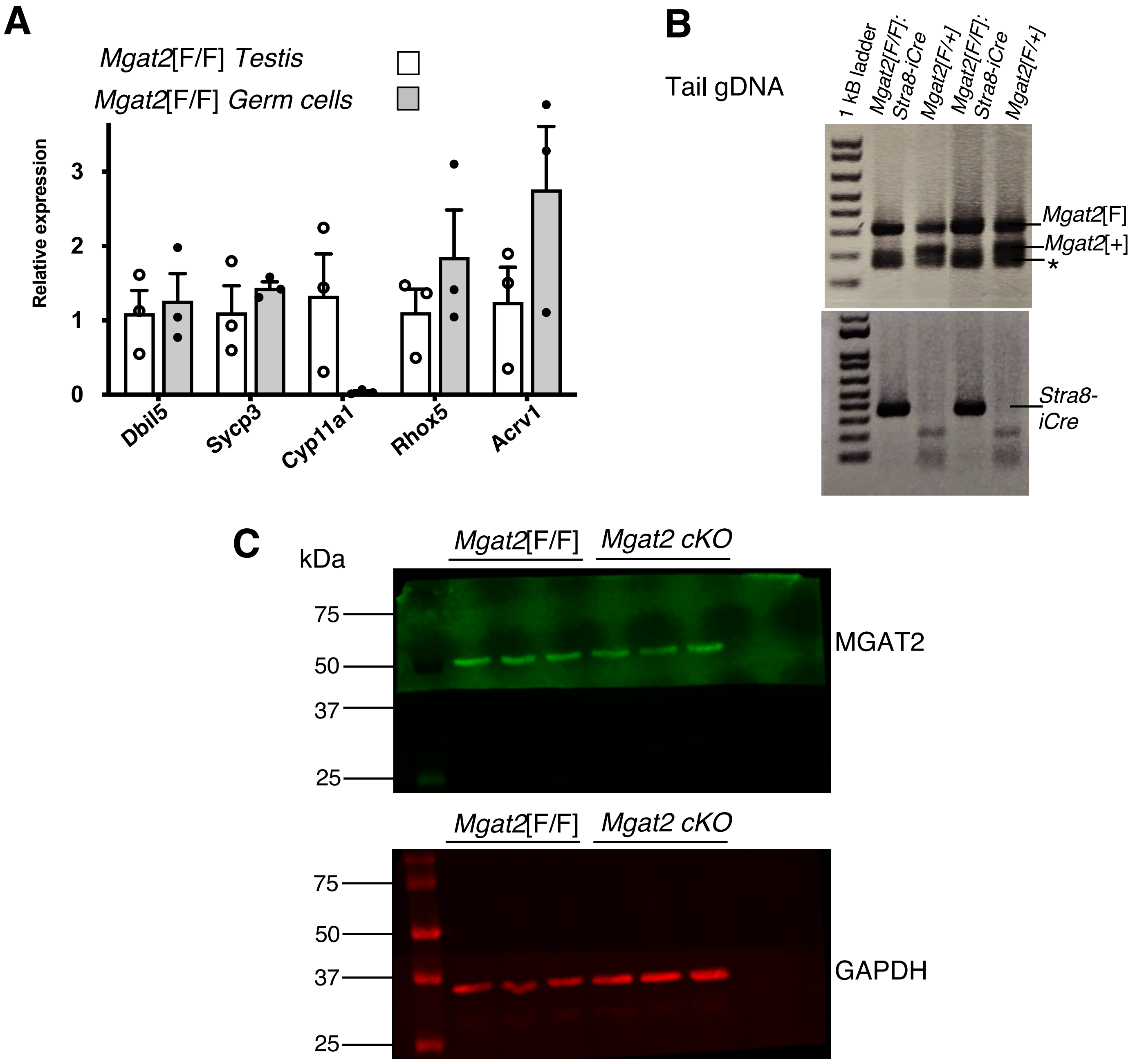

Figure S1. Germ cells enrichment, genotyping and western analysis. (A) qRT-PCR of testis cell-type marker genes expressed in 22-day *Mgat2*[F/F] testis versus enriched germ cells. Cell-type genes represented round spermatids (*Dbil5*), spermatocytes (*Sycp3*), Leydig cells (*Cyp11a1*), Sertoli cells (*Rhox5*) and spermatids (*Acrv1*). (B) PCR of tail genomic DNA (gDNA) to determine *Mgat2* genotypes as marked. *Mgat2*[F], 327 bp; *Mgat2*[+], 217 bp; *Stra8*-iCre, 380 bp; *, non-specific product. (C) Western blots quantitated to generate Fig. 1E. The gel was cut below the MGAT2 band and the top portion used to detect MGAT2, the bottom portion to detect GAPDH. Imaging was performed by LICOR Fc Odyssey.

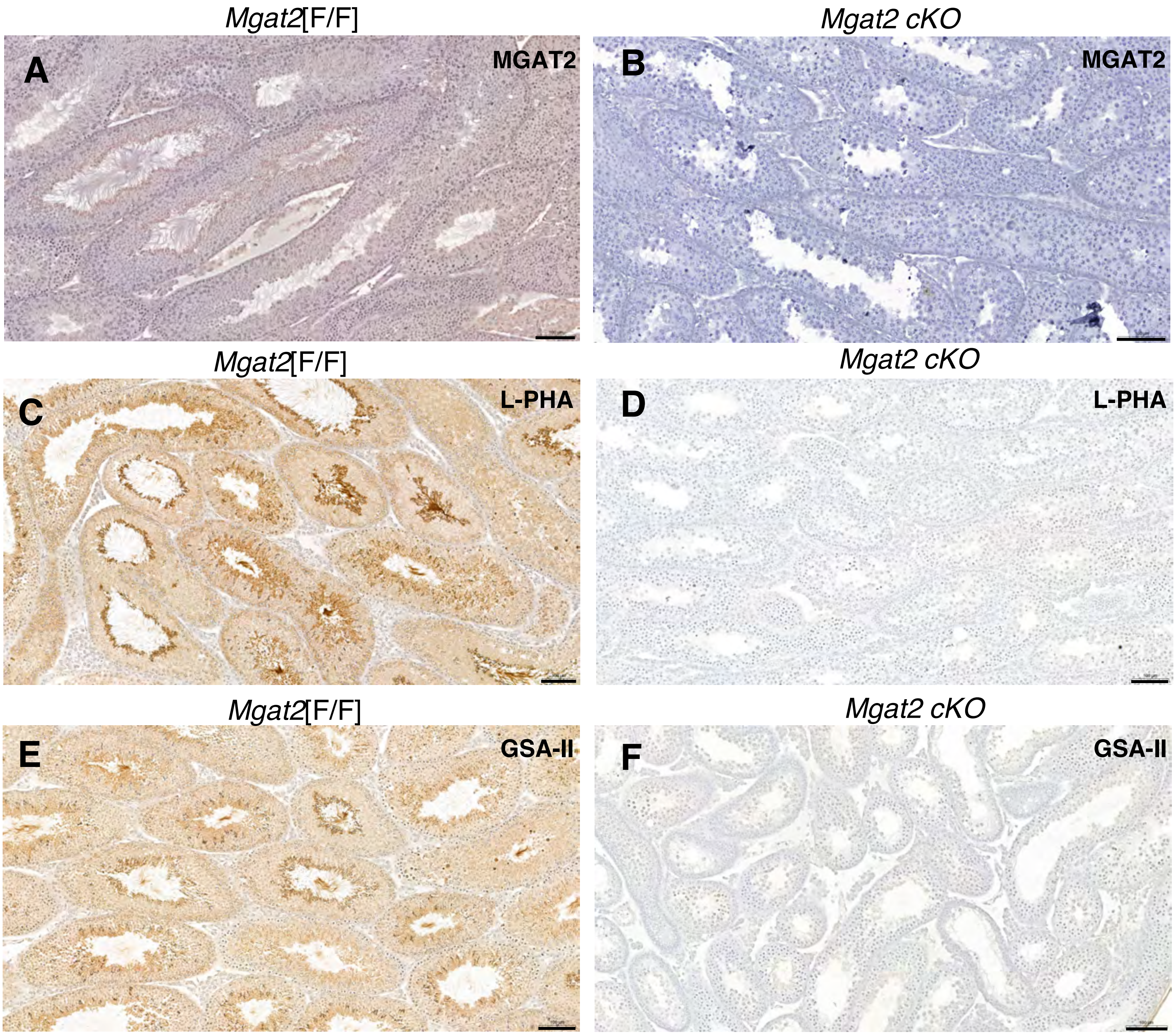

Figure S2. *Mgat2* cKO germ cells do not express MGAT2 or complex N-glycans. (A, C, E) Images of testis sections showing that *Mgat2*[F/F] testis tubules contained germ cells that expressed MGAT2 and complex N-glycans that bound L-PHA and GSA-II. (B, D, F) Images of testis sections showing that *Mgat2* cKO germ cells did not express MGAT2 nor complex N-glycans that bound L-PHA or GSA-II. Scale bars 100 μm.

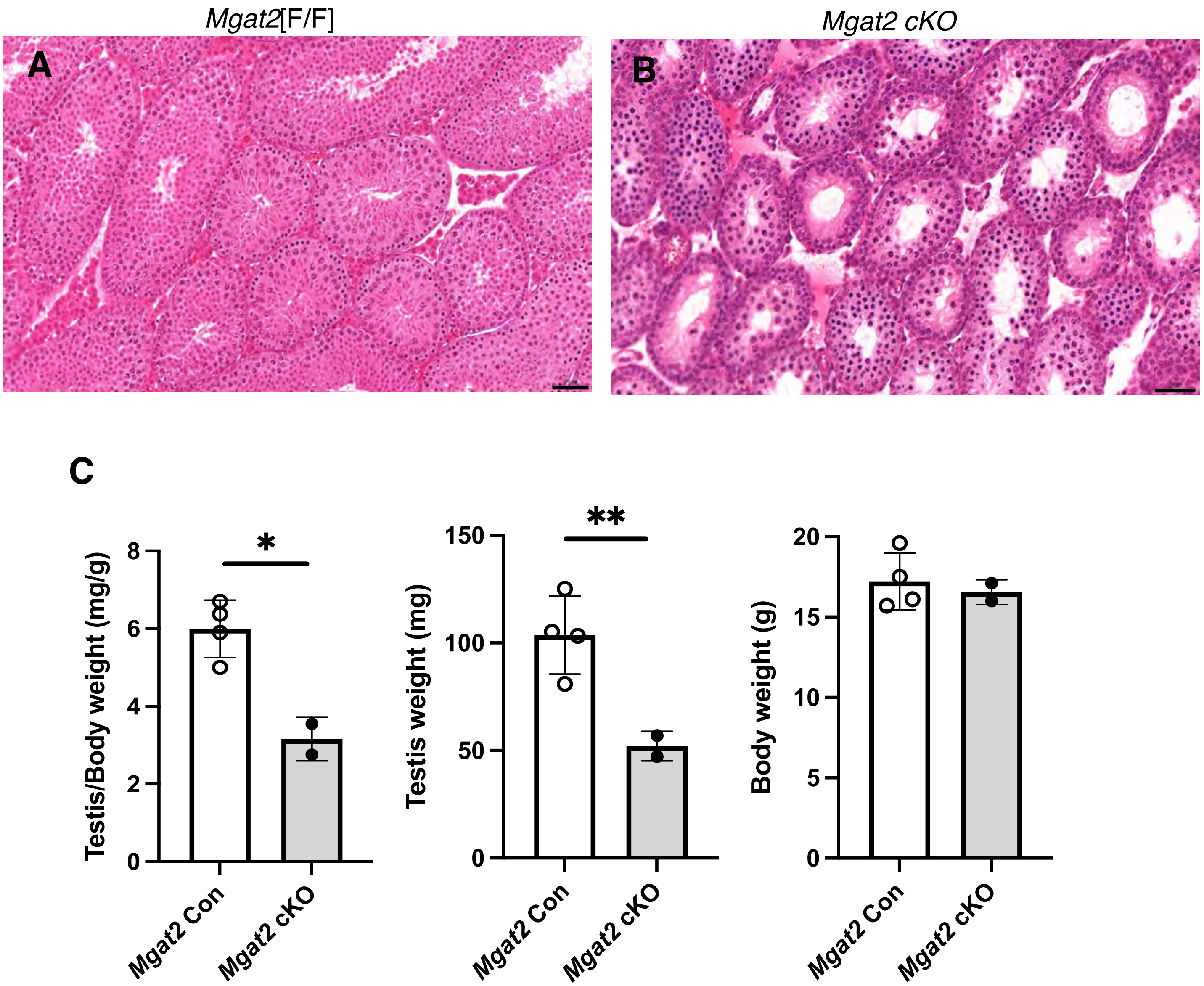

Figure S3. *Mgat2* cKO testis tubules at 4 weeks. (A, B) Deletion of *Mgat2* by *Stra8*-iCre resulted in marked disruption of spermatogenesis at 4 weeks. Scale bars 50 µm. (C) Testis and body weights of *Mgat2*[F/F] and cKO males at 4 weeks. Testis weight but not body weight was reduced in males lacking MGAT2 in germ cells.

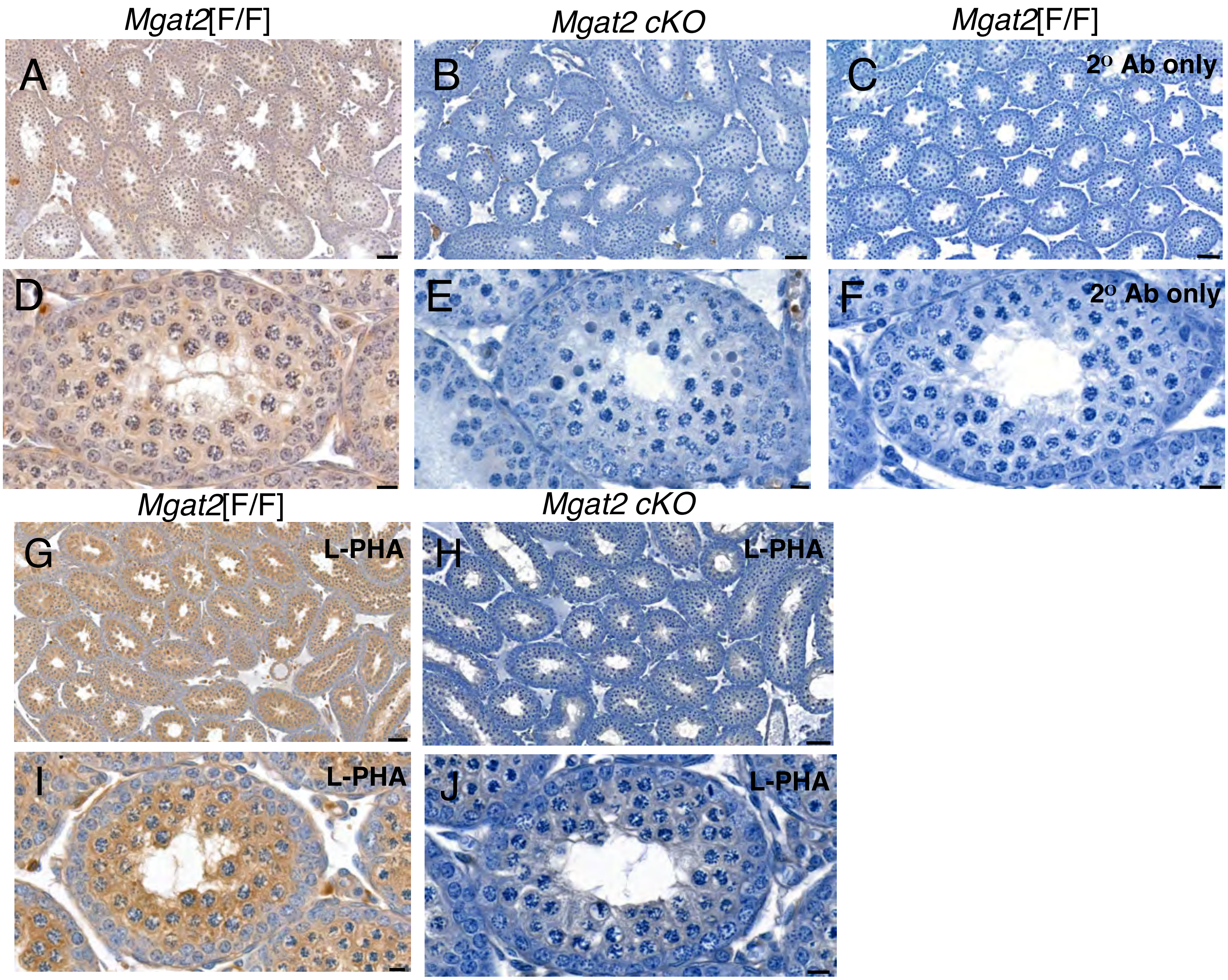

Figure S4. Mgat2 cKO germ cells do not express MGAT2 or complex N-glycans at 15 days.
(A, B, C) Representative images of sections from 15-day testes. MGAT2 was detected only in *Mgat2*[F/F] testes treated with both primary and secondary Abs. Scale bars 50 µm. (D, E, F) Single testis tubules from A, B, C respectively. Scale bars 10 µm. (G, H) Only *Mgat2*[F/F] germ cells bound L-PHA which detects complex N-glycans. Scale bars 50 µm. (I, J) A single testis tubule from G, H respectively. Scale bars 10 µm).

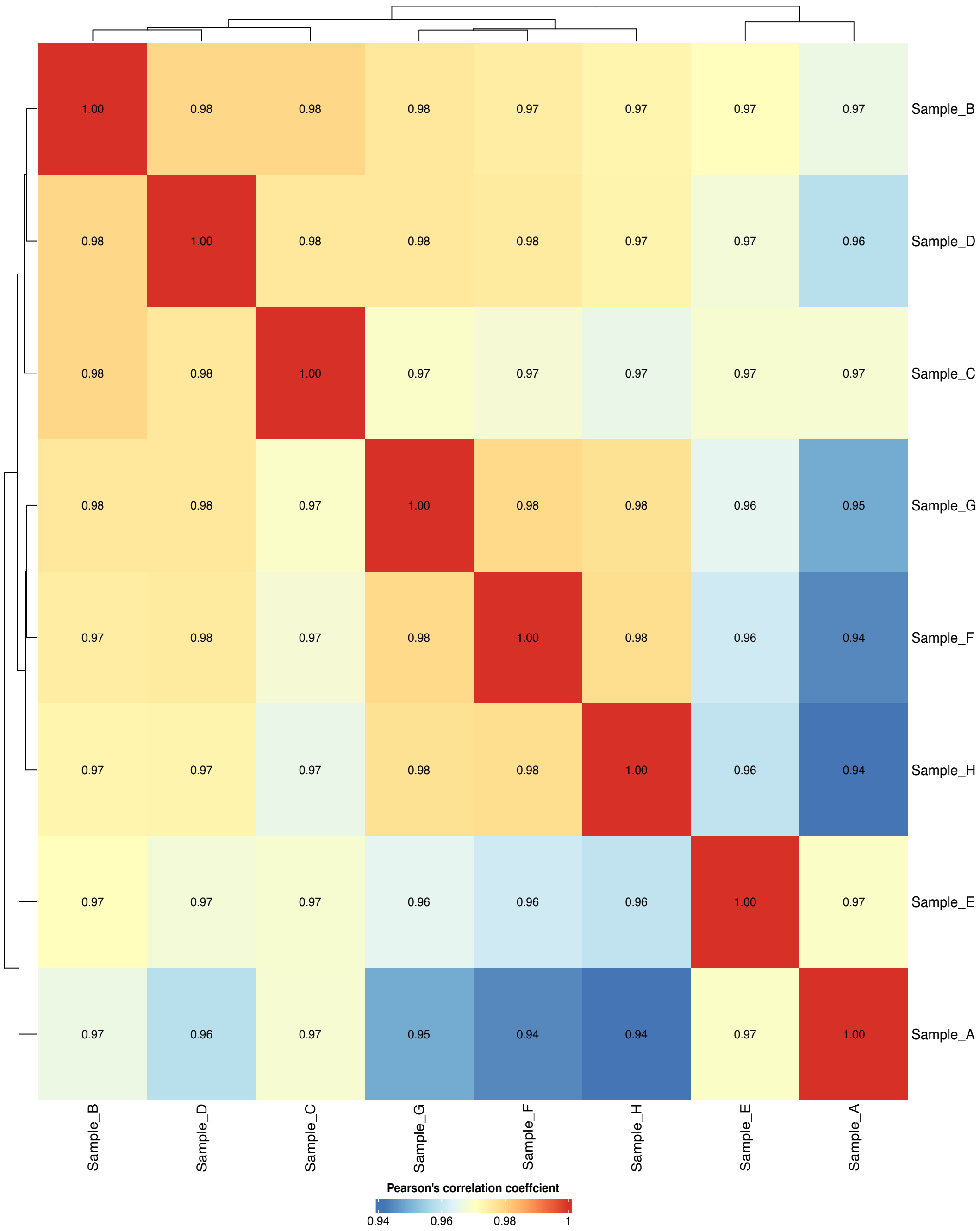

Figure S5. Pearson correlation coefficient for RNA-seq samples. The heatmap and color scale show the relationships between RNA-seq data from the 4 *Mgat2*[F/F] samples (A to D) and the 4 *Mgat2* cKO samples (E to H).

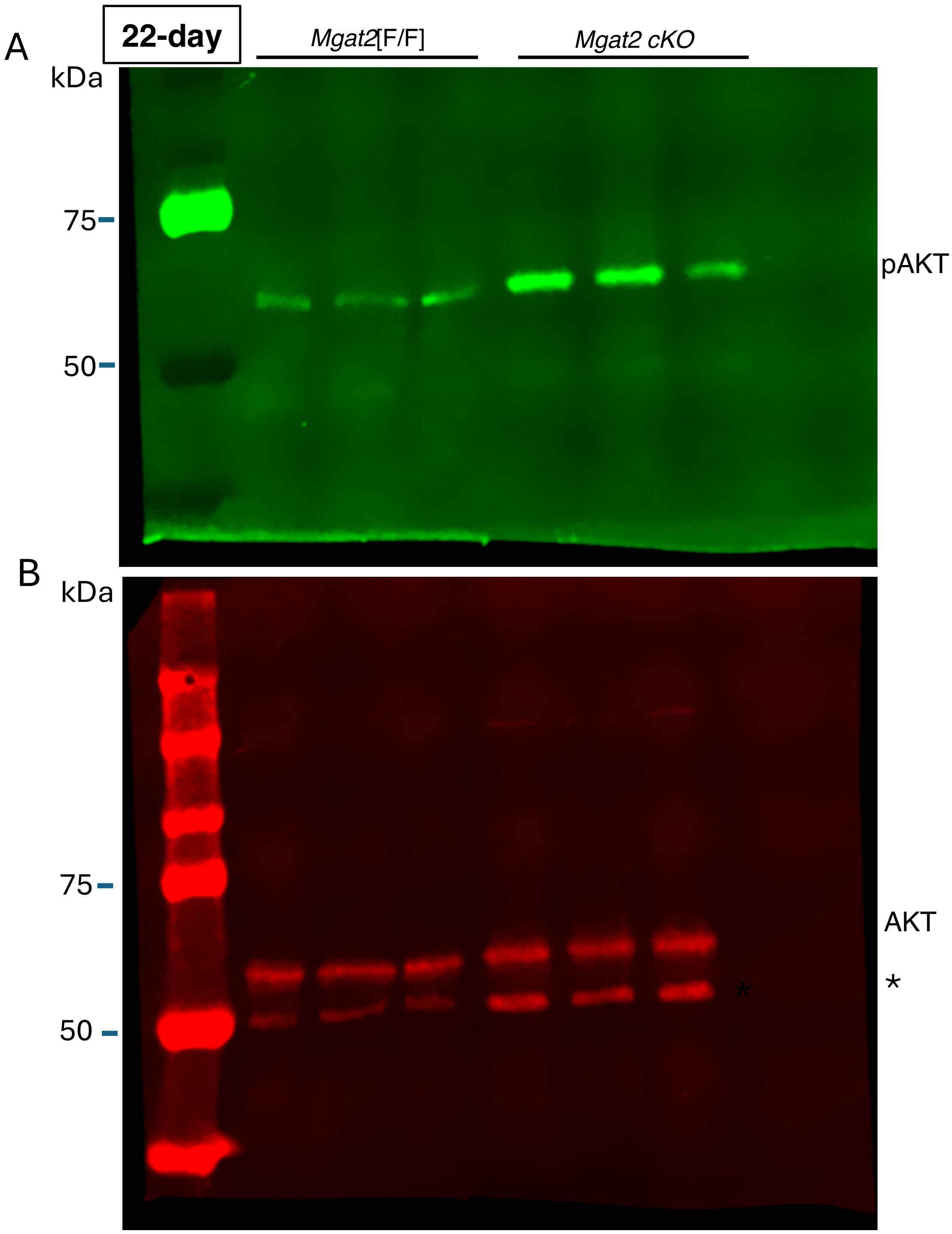

Figure S6. AKT signaling in 22-day *Mgat2*[F/F] versus *Mgat2* cKO germ cells.
(A, B) Full length gels from which the data in Fig. 8A were taken.
* non-specific band

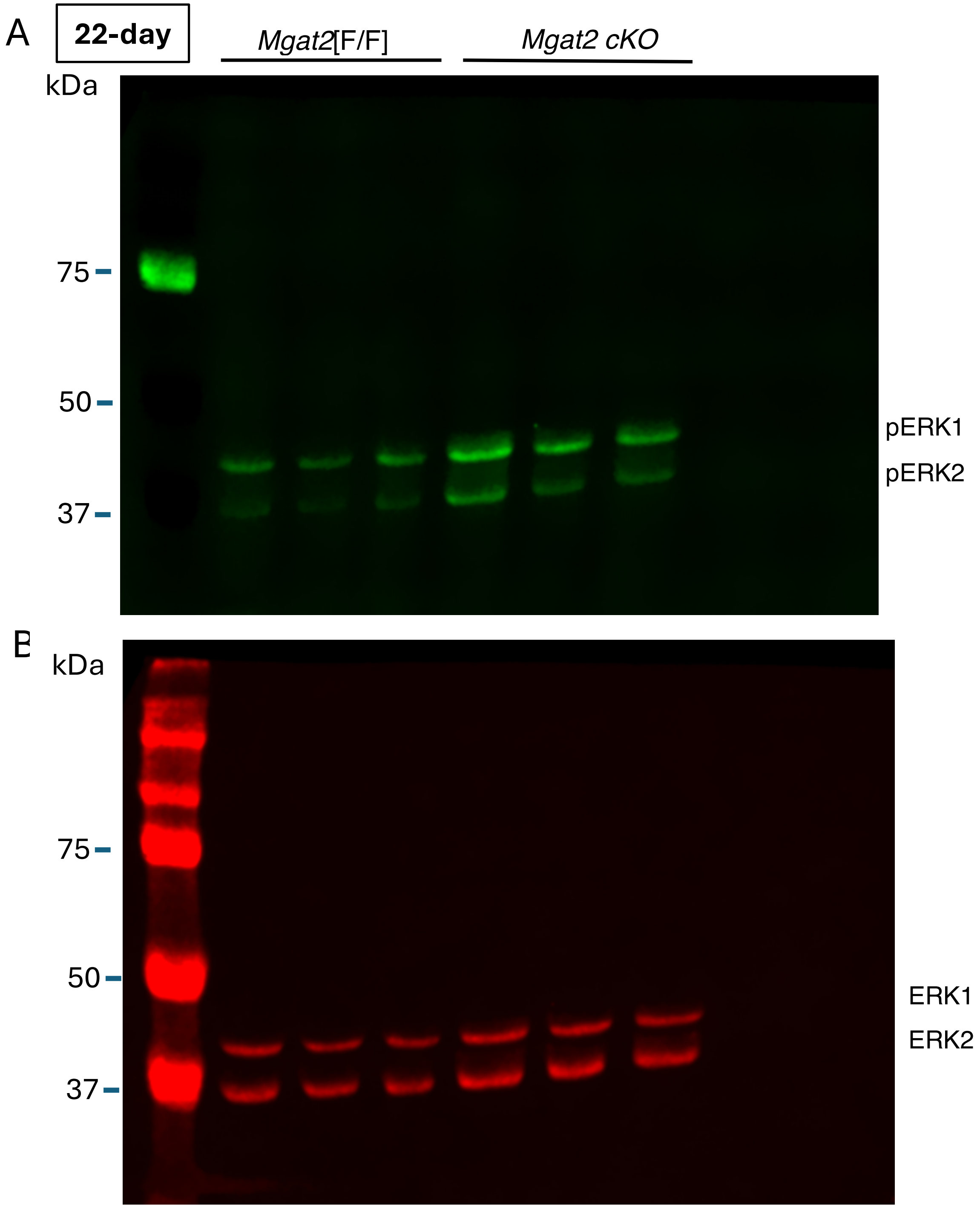

Figure S7. ERK signaling in 22-day Mgat2[F/F] versus cKO germ cells. (A, B) Full length gels from which the data in Fig. 8B were taken.

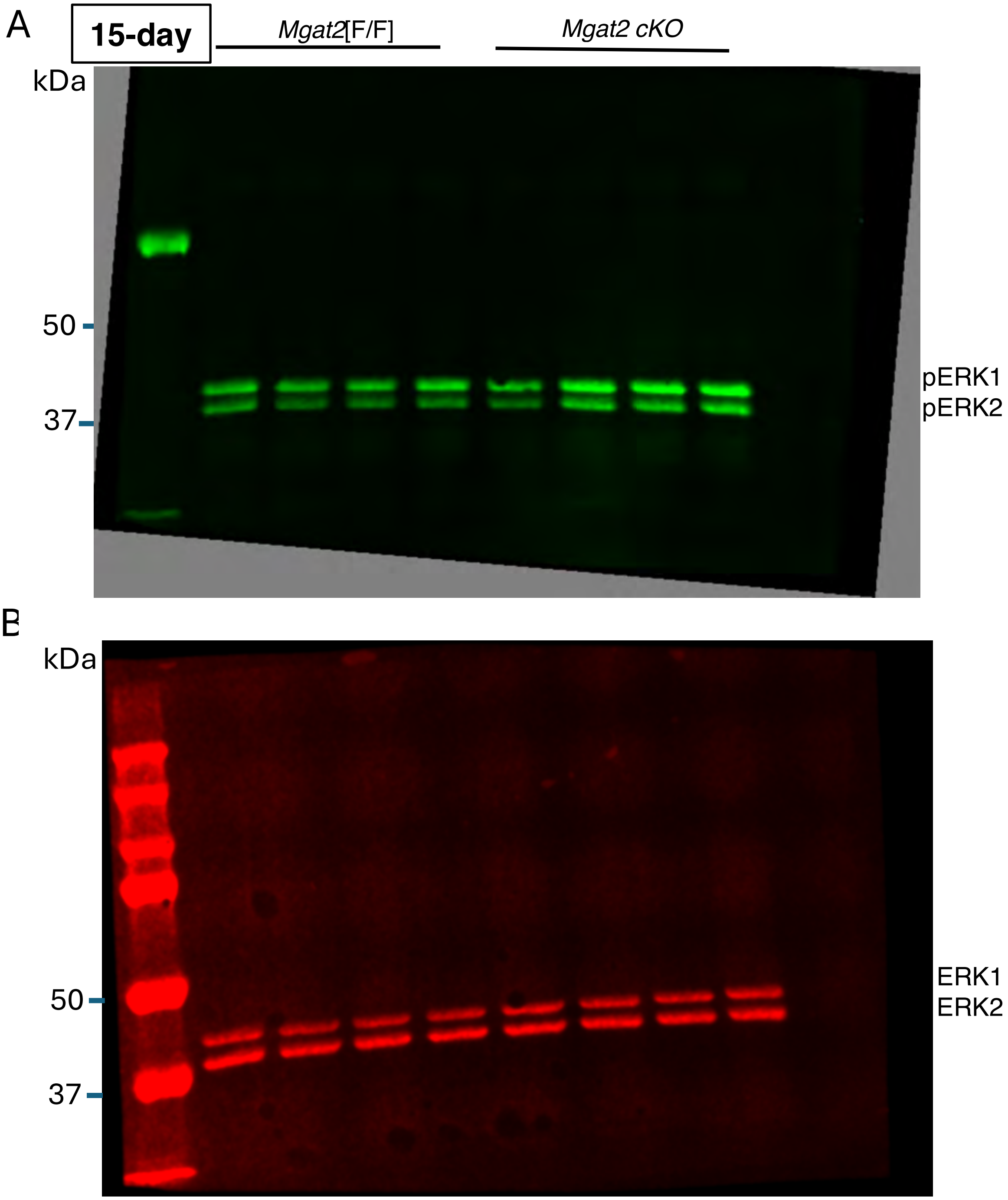

Figure S8. ERK signaling in 15-day *Mgat2*[F/F] versus cKO germ cells. (A, B) Full length gels from which the data in Fig. 8C were taken.

Table S1. RNA quality for RNA-seq.

Mouse Genotype	Body (g)	Left testis (mg)	Right testis (mg)	Total RNA (ug)	RNA quality (RQN)
Mgat2[F/F]					
A	6.8	14	14.1	42	10
B	8.1	13.1	13.5	40	10
C	8.2	13.8	14.4	36	10
D	7.9	11	12.3	38	10
Mgat2[F/F]: *Stra8*-iCre					
E	8	13.1	14.5	24	10
F	7.8	12.1	12.6	31	10
G	7.6	11.4	12.4	28	10
H	7.1	11.1	11.7	33	10

Control and *Mgat2* cKO males were sacrificed at 15 days, weighed, testes dissected, weighed and germ cells prepared. RNA was extracted with RNAZol as described in Experimental Procedures. These RNA samples were processed for RNA-seq.

Table S2. Top down-regulated genes in *Mgat2* cKO germ cells.

Gene	Gene Name	Fold Decreased
Cfap58	cilia and flagella associated protein 58	13.6
Cfap221	cilia and flagella associated protein 221	11.2
Prok2	prokineticin 2	7.9
Igsf21	immunoglobulin superfamily, member 21	7.2
Cfap44	cilia and flagella associated protein 44	6.2
Fam3d	FAM3 metabolism regulating signaling molecule D	6.0
Rp1	RP1 axonemal microtubule associated	5.8
Tmem30c	transmembrane protein 30C	5.3
Daw1	dynein assembly factor with WDR repeat domains 1	5.2
Ccdc41	centrosomal protein 83	5.2
Lrrc71	leucine rich repeat containing 71	5.0
Catspere1	cation channel sperm associated auxiliary subunit epsilon 1	4.9
Cdhr4	cadherin related family member 4	4.9
Myorg	myogenesis regulating alpha-galactosidase	4.6
Plcd4	phospholipase C delta 4	4.6
Arhgap33os	Rho GTPase activating protein 33, opposite strand	4.4
Dnaaf6	dynein axonemal assembly factor 6	4.4
Piwil1	Piwi-like RNA-mediated gene silencing 1	4.2
Rfx4	regulatory factor X4	4.1
Svop	SV2 related protein	4.0

Table S3. Most significantly down-regulated genes in *Mgat2* cKO germ cells.

Gene	Gene Name	Padj Value
Catsperg2	cation channel sperm associated auxiliary subunit gamma 2	6.4E-10
Pcsk4	proprotein convertase subtilisin/kexin type 4	1.9E-09
Lrrc71	leucine rich repeat containing 71	8.8E-09
Gstt2	glutathione S-transferase theta 2	9.7E-09
Cfap74	cilia and flagella associated protein 74	2.5E-07
Ccdc39	coiled-coil domain 39 molecular ruler complex subunit	2.6E-07
Adad2	adenosine deaminase domain containing 2	4.2E-07
Mapk15	mitogen-activated protein kinase 15	4.4E-07
Ptpn20	protein tyrosine phosphatase non-receptor type 20	1.3E-06
Tmem30c	transmembrane protein 30C	1.9E-06

Table S4. Molecules in IPA Network 2 in Fig.7*A*.

**ABO**↓	**ADGB**↓	AKT1↑	**BIN2**↓
CCL24	**Ccl27a**↓	CCR10	chemokine
**CHFR**↓	**CKLF**↓	**Cyct**↓	cytochrome C
cytochrome-c oxidase	**ENO3**↓	FOXJ1↓	glutathione peroxidase
GST	**GSTT2/GSTT2B**↓	Histone h3	HMBS↑
MALAT1	**MAPK15**↓	**MATCAP2**↓	NFE2L2↑
PAQR3↓	**PHF1**↓	**RHBDD3**↓	SHC1↑
**SLC12A5**↓	**SPAG8**↓	**SPATA33**↓	STING1↓
TP53I3	YAP1↑	**ZBTB32**↓	

Bolded are transcripts significantly down (green arrow) or up (red arrow) in *Mgat2* cKO germ cells and present in our RNA-seq dataset. Arrows indicate direction of activity for non-bolded molecules; red, activated; green, inhibited.

Tables S5. Molecules in IPA Network 1 in Fig. 7*B*.

**C18orf54**	**CCAR2**	**CCHCR1**	**CCP110**
**COL16A1**	**DNAJB1**	**DVL1**	**ENKD1**
ERK1/2	**FAM184A**	**FAM228A**	**FANCG**
**FBRS**	**FCGR2A**	**GLYCTK**	**GNAO1**
**GOLGA2**	Integrin	**LRRC61**	**MIF4GD**
**MORN5**	**PCGF6**	**PHF1**	PLC
**PLCD4**	**PNMA1**	**PNMA8A**	RAP1
**SPAG8**	**STAMBP**	**Tcam1**	**TCEA2**
**TKFC**	tyrosine kinase	**WDR62**	

Bolded are transcripts significantly up (red) in Control germ cells. and present in our RNA-seq dataset. Non-bolded are not significantly different in *Mgat2* cKO compared to Control germ cells.

Tables S6. Molecules in IPA Network 2 in Fig. 7*C*.

activin (family)	AKT
**CHFR**	CRL E3 ubiquitin ligase complex:COMMDs:CCDC22:DCUN1Ds
CRL E3 ubiquitin ligases	**DPP8**
E3 ligases in proteasomal degradation	E3:K48-iquitinated substrate
**FKTN**	IFT B
**IFT22**	**IFT70A**
**KHDRBS3**	**LRGUK**
PRKAA	**PROK2**
**SLC9A5**	**TMPRSS12**
anterograde IFT trains	**ARMT1**
CRL E3 ubiquitin ligase:CAND1	CRL E3 ubiquitin ligase:COMMDs:CCDC22
**DYNC2H1**	**DYNC2I1**
**FBXO32**	**FBXW5**
IFT B*	**IFT172**
**KBTBD6**	**KCTD7**
**MAN2C1**	**POMT1**
**RIN1**	**SENP2**
**TTC21B**	

Bolded are transcripts significantly up (red) in Control germ cells. and present in our RNA-seq dataset. Non-bolded are not significantly different in *Mgat2* cKO compared to Control germ cells.

Table S7. Primers used in qRT-PCR and genotyping

Gene	Forward 5'- 3'	Reverse 5'- 3'
**Primers for qRT-PCR**		
Mgat2	GAATACCCTGACTCCTTCGG	CTGGGGCTAAGTAGTGGTCC
Mgat1	GGGCTTGTATTCGTCCAGAA	CTTCACCCAGTTGGACCTGT
Sycp3	CATTCTGGGAAATCTGGGAAGCCACCT	CCAGCATATTCTGTACTTCACCTCCAAC
Acrv1	TGAAGTTTCGGGTGACGAAGCAGGT	GCTGGGAGTTTTGAGTGGTGCATAC
Dbil5	GTACAGCTTTTACAAACAGGCCACCC	CCACTTTAGCAATGTAGATCCTCATGGC
Cyp11a1	GTGGACCCCAAGGATGCGTCGATACTCTTC	ACCTCTTGGTTTAGGACGATTCGGTC
Rhox5	TCAAGGAAGACTCGGAAGAACAGCAT	CACTATCCTTGTCCCCATCACCCATA
Actin	CGGTTCCGATGCCCTGAGGCTC	TGTCAGCAATGCCTGGGTACATGGTGGT
Sox9	GTGGCAAGTATTGGTCAA	GAACAGACTCACATCTCT
Gapdh	AAGGTCATCCCAGAGCTGAA	CTGCTTCACCACCTTCTTGA
**Primers for genotyping**		
Mgat2[Flox/+]	GCTGCTAGGTGGGTGCTGGA	TTCGAGGCCAGCCTGGTTTA
Stra8-iCre	GACAGGGCTGTGATTGGTTC	CACAGTCAGCAGGTTGGAGA
Cre	GGACATGTTCAGGGATCGCCAGGCGT	GCATAACCAGTGAAACAGCATTGCTG
